# Functional Genomic Characteristics of Marine Sponge-Associated *Microbulbifer spongiae* MI-G^T^

**DOI:** 10.3390/microorganisms13081940

**Published:** 2025-08-20

**Authors:** Nabila Ishaq, Qianqian Song, Micha Ilan, Zhiyong Li

**Affiliations:** 1 Marine Biotechnology Laboratory and State Key Laboratory of Microbial Metabolism, School of Life Sciences and Biotechnology, Shanghai Jiao Tong University, Shanghai 200240, China; nabilaishaq@sjtu.edu.cn (N.I.); sqqsjc@sjtu.edu.cn (Q.S.); 2 School of Zoology, Faculty of Life Sciences, Tel-Aviv University, Tel-Aviv 6997801, Israel; milan@tauex.tau.ac.il; 3 Hainan Research Institute, Shanghai Jiao Tong University, Sanya 572025, China; 4 Joint International Research Laboratory of Metabolic & Developmental Sciences, Shanghai Jiao Tong University, Shanghai 200240, China

**Keywords:** complete genome sequencing, comparative genomics, environmental stress, metabolic features

## Abstract

The genus *Microbulbifer* comprises a group of marine, gram-negative bacteria known for their remarkable ability to adapt to a variety of environments. Therefore, this study aimed to investigate the genetic diversity and metabolic characteristics of *M. spongiae* MI-G^T^ and three *Microbulbifer* reference strains by genomic and comparative genomic analysis. Compared to free-living reference strains, the lower GC content, higher number of strain-specific genes, pseudogenes, unique paralogs, dispensable genes, and mobile gene elements (MGEs) such as genomic islands (GIs) and insertion sequence (IS) elements, while the least number of CAZymes, indicates that *M. spongiae* MI-G^T^ may be a facultative sponge-symbiont. Comparative genomic analysis indicates that *M. spongiae* MI-G^T^ possesses a plasmid and a higher number of strain-specific genes than *Microbulbifer* reference strains, showing that *M. spongiae* MI-G^T^ may have acquired unique genes to adapt sponge-host environment. Moreover, there are differences in the functional distribution of genes belonging to different COG-classes in four *Microbulbifer* strains. COG-functional analysis reveals a lower number of strain-specific genes associated with metabolism, energy production, and motility in *M. spongiae* MI-G^T^ compared to *Microbulbifer* reference strains, suggesting that sponge-associated lifestyle may force this bacterium to acquire nutrients from the sponge host and loss motility genes. Finally, we found that several proteins associated with oxidative stress response (sodC, katA, catA, bcp, trmH, cspA), osmotic stress response (dsbG, ampG, amiD_2, czcA, czcB, and corA), and tolerance to biotoxic metal proteins (dsbG, ampG, amiD_2, czcA, czcB, and corA) are absent in *M. spongiae* MI-G^T^ but present in *Microbulbifer* reference strains, indicating that *M. spongiae* MI-G^T^ live in a stable and less stress environment provided by the sponge host than free-living *Microbulbifer* strains. Our results suggest *M. spongiae* MI-G^T^ exhibits gene characteristics related to its adaptation to the sponge host habitat, meanwhile reflecting its evolution towards a sponge-associated lifestyle.

## 1. Introduction

Sponges (phylum Porifera) are the oldest multicellular animals (metazoans) and are ubiquitous in marine systems [1]. In line with the holobiont concept, sponge-associated microorganisms may contribute to many aspects of the sponge’s physiology and ecology [2,3,4]. For example, sponge-associated bacteria are thought to be essential for host health, nutrition, defence against pathogens, removal of by-products, and synthesis of bioactive compounds [5,6]. Recent studies have suggested that sponge-associated bacteria have the capacity to metabolize carbon (e.g., polysaccharides), sulfur, nitrogen, and possess diverse eukaryotic-like proteins (ELPs). Moreover, Engelberts et al. found that sponge-associated microbes can metabolize carbon, nitrogen, and sulfur, and synthesize essential B-vitamins [7,8,9,10]. Functional capacities of sponge-associated bacteria have been increasingly recognized, but their genomic characteristics remain largely unexplored. Exploration of sponge-associated bacteria offers exciting opportunities for new discoveries and evolution of novel bacterial strains.

The advancement of high-throughput sequencing technologies and comparative genomic approaches has significantly enhanced our understanding of bacterial genomes, genetic diversity, evolution, and functional capabilities [11,12]. Genome analysis also provides an ideal strategy to identify differences between species within the same genus [13,14]. Previous genomic studies reveal that bacterial strains inhabiting different environments possess various genes responsible for drug resistance, heavy metal resistance, and general stress response to survive in fluctuating and challenging environmental conditions [15]. Moreover, bacterial mobile gene elements (MGE), such as plasmids, genomic islands (GIs), insertion sequences (ISs), and CRISPR-Cas systems, are important players in bacterial evolution and contribute to the prevalence of phenomena of horizontal gene transfer (HGT) [16]. Thus, MGEs are pivotal in shaping bacterial genomes through their ability to move within a host’s genome and jump between different bacterial genomes [10,17]. These elements are key drivers of genome evolution through their roles in potentiating gene gain and loss. So, mobilomes profoundly influence bacterial fitness and survival by changing adaptability and facilitating rapid changes in gene content [18]. This change can contribute to the genetic adaptation to new environments and the emergence of divergent bacterial populations that may produce evolutionary distinct species [19].

*Microbulbifer* is a genus of *Gammaproteobacteria* that is widespread in aquatic environments. The ubiquitous distribution of *Microbulbifer* bacteria forces them to evolve specific biological characteristics to adapt to particular habitats [20]. These bacteria also play significant role in the degradation of organic matter and contribute to the cycling of nutrients [21,22,23,24]. For example, *M. chitinilyticus*, *M. harenosus*, and *M. mangrove* can degrade a wide variety of carbohydrates e.g., cellulose, starch, and xylan. Previously, carbohydrate-active enzymes, i.e., GH (glycoside hydrolase), CBM (carbohydrate binding modules), CE (carbohydrate esterase), GT (glycosyltransferase), AA (auxiliary activities), and PL (polysaccharide lyases) were identified in the genome of *Microbulbifer* sp. ALW1 [22,25,26]. However, genetic knowledge of *Microbulbifer* strains is fairly limited. Thus, further genome-based studies are required to comprehensively explore the survival mechanism of *Microbulbifer* group members, particularly how novel *Microbulbifer* species evolve and survive in their particular habitat.

To date, only a few studies have been conducted to reveal genome plasticity and evolution of sponge-associated bacterial species [18,27], particularly, no systematic functional genomic comparison between closely related sponge-associated bacteria and free-living bacteria has been conducted till now. Therefore, this study aims to provide comprehensive insights into the genomic diversity and metabolic characteristics that may lead to the evolution of the novel bacterial strain *Microbulbifer spongiae* MI-G^T^, recently isolated from the sponge *Diacarnus erythraeanus* [28]. Genomic and comparative genomic analysis of *M. spongiae* MI-G^T^ with three *Microbulbifer* reference strains was conducted to identify distinct genomic features such as mobile gene elements, carbohydrate-active enzymes (CAZymes), stress response elements, and host-bacteria metabolic interaction-related features. Finally, the genes of four *Microbulbifer* strains were functionally classified according to COG-functional classification to reveal overall functional genetic diversity.

## 2. Materials and Methods

### 2.1. Sample Collection, Genome Sequencing, and Assembly

Marine sponge *Diacarnus erythraeanus* was collected from mesophotic reefs, in front of the interuniversity institute for marine sciences in Eliat, Israel (29°30′06.6″ N 34°54′59.9″ E) in December 2020. A small *D. erythraeanus* subsample (0.2 × 0.2 cm) was sonicated for 2 min in 1 mL 1× PBS buffer solution (KH_2_PO_4_ 1.76 mM; KCl 2.67 mM; NaCl 136.89 mM; Na_2_HPO_4_ 8.1 mM) (Sangon Biotech, Shanghai, China). After10-fold serial dilutions, 100 µL samples were spread on ZoBell marine agar plates (AC12065; ACMEC); individual colony was purified by repeated re-streaking after 20 days of incubation. Upon screening of all sponge-associated microbes, a novel *M. spongiae* MI-G^T^ was cultured and identified using a method previously described [28,29], and stored in 30% (*v*/*v*) glycerol suspension at −80 °C.

Genomic DNA of *M. spongiae* MI-G^T^ was prepared using a TIANamp bacterial genomic DNA extraction kit (Tiangen Biotech, Beijing, China), according to manufacturer’s instructions. DNA concentration was quantified using a Qubit 2.0 Fluorometer (Thermo Fischer Scientific, Waltham, MA, USA). DNA integrity and purity were detected by Agarose Gel Electrophoresis (Concentration of Agarose Gel: 0.5%, Voltage:150 V, Electrophoresis Time: 40 min) (Figure S1). The whole genome sequencing of high-quality genomic DNA of strain MI-G^T^ was performed using a DNBSEQ (BGI) and Nanopore (ONT) platform at the Beijing Genomics Institute (BGI, Shenzhen, China) [28,30]. The methodological details are as follows: 1 ug genomic DNA was fragmented by Covaris. Fragmented genomic DNA was selected using the Agencourt AMPure XP-Medium kit to an average size of 200–400 bp. DNA fragments were end-repaired and then 3′-adenylated. After that, fragments were amplified and formatted into a final library. The library was qualified by quality control assessment (QC). The qualified libraries were sequenced by BGISEQ-500. Raw sequenced data was filtered and trimmed using SOAPnuke v 1.5.6 (Parameter: -l 20 -q 40% -n 10% -d), and Porechop v 0.2.4 with default methods [31,32]. Draft genomic unitigs were assembled using Canu v1.5 with the following parameters: estn = 24, npruseGrid = 0, corOvlMemory = 4 (https://github.com/marbl/canu/releases, accessed on 16 February 2022). After that, GATK (https://gatk.broadinstitute.org/hc/en-us, accessed on 29 January 2022) was used to make single-base corrections to improve the accuracy of the genome sequences [33].

### 2.2. Genome Annotation and Analysis

Gene prediction was performed on the assembled genome of *M. spongiae* MI-G^T^ by GeneMarkS-2+ (http://topaz.gatech.edu/GeneMark/genemarks2.cgi) [34]. Barrnap v0.9 [35] was used to find rRNAs, tRNAscan-SE version 1.3.1 [36] was used to predict the area of tRNA and its secondary structure, and Rfam v15.0 (http://rfam.org/) was used to compare with the Rfam database and get sRNAs [37]. Island viewer 4 (https://www.pathogenomics.sfu.ca/islandviewer/) was used for genomic island analysis with Island Pathe-DIOMB, SIGI-HMM, and Island Picker method [38]. Insertion sequence (IS) finder (https://isfinder.biotoul.fr/about.php) was used to identify insertion sequence elements [39]. CRISPRCasFinder (https://crisprcas.i2bc.paris-saclay.fr/CrisprCasFinder/Index) program was used for the detection of CRISPRs and cas-genes [40]. A graphical map of the circular genome was generated using Circos (https://mycircos.iric.ca) [41].

Functional annotation of the genome was accomplished by analysis of protein sequences. Genes were aligned with databases to obtain their corresponding annotations; only the best-hit Blast alignment results were chosen as gene annotations to ensure the biological meanings. Functional annotation was completed by blasting genes with different databases using Diamond (software, v0.8.24) [42]. Seven databases i.e., Kyoto Encyclopedia of Genes and Genomes (KEGG, version 89.1), Clusters of Orthologous Groups (COG, version 2020-11-25), Gene Ontology (GO, release_2-2019_07_01), Non-Redundant Protein Database databases (NR, version 2021_11_17), Swiss-Prot (version, release-2021_04), EggNOG (version 5.0), and TrEMBL were used for gene function annotation [43,44,45,46,47]. Four databases such as Virulence Factors of Pathogenic Bacteria (VFDB, version 2021-11-25), Antibiotic Resistance Genes Database (ARDB, version 1.1), Type III secretion system effector proteins (T3SS, version 1.0), and Carbohydrate-Active enZYmes Database (CAZy, version 2021-10-13) [48,49,50] were used for pathogenicity and drug resistance analysis (Figure S3). We used hmmscan version 3.4 and dbCANv3 (https://bcb.unl.edu/dbCAN2/, accessed on 25 November 2024) web-resource to annotate CAZyme [51].

### 2.3. Comparative Genomic Analysis

*M. spongiae* MI-G^T^ genome was compared to the genomes of three *Microbulbifer* reference strains that were isolated from different marine environments, i.e., *M. hydrolyticus* IRE31^T^ (marine pulp mill effluent), *M. thermotolerance* DAU221^T^ (deep-sea sediments), and *M. variabilis* ATCC700307^T^ (marine algae). Synteny of *M. spongiae* MI-G^T^ and three *Microbulbifer* reference strains was performed using MUMmer version 4.0 (http://mummer.sourceforge.net/), and BLAST (https://blast.ncbi.nlm.nih.gov/Blast.cgi) (e-value < = 1 × 10^−5^, identity > = 85%) [52]. Core and pan genes of *M. spongiae* MI-G^T^ and three *Microbulbifer* reference strains IRE31^T^, DAU221^T^, and ATCC700307^T^ were clustered by CD-HIT (software v4.8.1) with a threshold of 50% pairwise identity and 0.7 length difference cutoff in amino acids [53]. BLAST coverage ratio (BCR) of genes from the gene pool and query sample was calculated separately. If BCR values from reference and query samples are smaller than the setting value and the gene from the reference is not homologous with queries, then the gene from the query genome is added to the gene pool. Gene family was constructed by genes of *M. spongiae* MI-G^T^ and three *Microbulbifer* reference strains mentioned above, integrating multi software: align the present sequence in BLAST, eliminate the redundancy by solar, and carry out gene family clustering treatment for alignment results with Hcluster_sg software [54]. Then, we convert the alignment results of proteins into those of multiple sequence amino acids in the CDS area, after multiple sequence alignments with clustered gene families by using Muscle software version 3.8.31 [55]. Phylogenomic analysis was performed by uploading genome sequence data of *M. spongiae* MI-G^T^ and other closely related *Microbulbifer* strains to the Type Strain Genome Server (https://tygs.dsmz.de/) [56]. Single-copy orthologous cluster protein sequences were extracted by Proteinortho (Software, v6.3.4), and a neighbor-joining (NJ) phylogenetic tree was constructed from these sequences by using MEGA version 11.0 [57]. Figure S2. illustrates the workflow that outlines the step-by-step process for functionally characterizing a sponge-associated novel bacterial strain.

## 3. Results

### 3.1. Genome Features of Microbulbifer Spongiae MI-G^T^ and Three Microbulbifer Reference Strains

**Figure 1:**
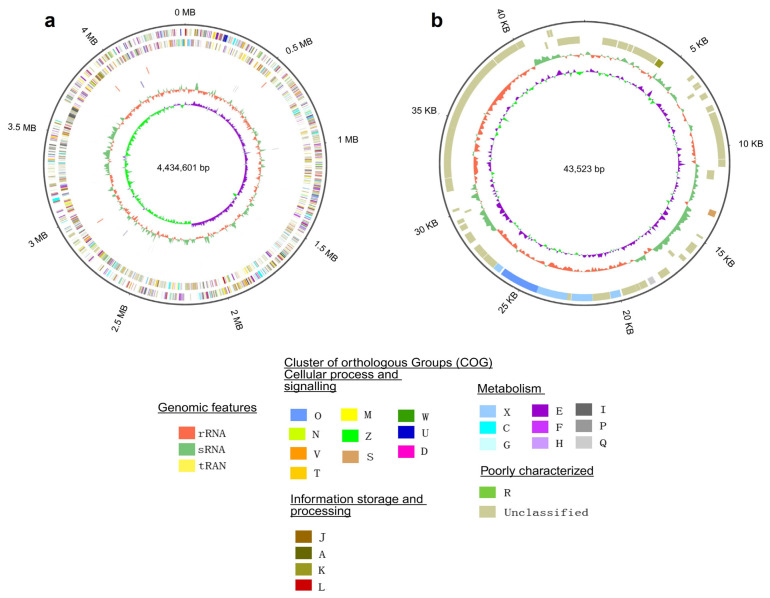
Circular representation of genome maps (**a**) Chromosome (CP098023.1), (**b**) Plasmid (CP098024.1) of *M. spongiae* MI-G^T^, generated using the Circos software (https://wlcb.oit.uci.edu/NG-Circos) (https://github.com/YaCui/NG-Circos) (Cui et al. 2020) [58]. (**a**) Chromosome (from outer to the inner): outermost circle depicts the genome size; forward strand gene, colored according to cluster of orthologous groups (COG) classification; reverse strand gene, colored according to cluster of orthologous groups (COG) classification; forward strand ncRNA; reverse strand ncRNA; repeat; GC content; GC skew. (**b**) Plasmid: (from outer to inner) genome size; forward strand gene, colored according to COG classification; reverse strand gene, colored according to COG classification; GC content; GC-SKEW. COG-classification includes the following categories: (O) Posttranslational modification, protein turnover, chaperons, (D) Cell cycle control, cell division, chromosome partitioning, (N) Cell motility, (M) Cell wall, membrane envelope, biogenesis, (Z) Cytoskeleton, (S) Function unknown, (T) Signal transduction mechanisms, (U) Intracellular trafficking, secretion, and vesicular transport, (V) Defense mechanisms, (W) Extracellular structures, (A) RNA processing and modification, (J) Translation, ribosomal structure and biogenesis, (K) Transcription, (L) Replication, recombination and repair, (F) Nucleotide transport, and metabolism, (C) Energy production and conversion, (G)Carbohydrate transport and metabolism, (H) Coenzyme transport and metabolism, (I) Lipid transport and metabolism, (E) Amino acid transport and metabolism, (P) Inorganic ion transport and metabolism, (Q) Secondary metabolites biosynthesis, transport and catabolism, (X) Mobilome: prophages, transposons, (R) General function prediction only.

The complete genome of *M. spongiae* MI-G^T^ (sponge holobiont) is composed of a single circular chromosome of 4,434,601 bp with 53.5% GC content, and a single circular plasmid of 43,523 bp with 52% GC content, respectively (Figure 1; Table 1). *M. spongiae* MI-G^T^ genome coverage is 267x, with 100% completeness and 0.56% contamination. The complete genome of three *Microbulbifer* reference strains, *M. hydrolyticus* IRE31^T^ (marine pulp mill effluent), *M. thermotolerance* DAU221^T^ (deep-sea sediments), and *M. variabilis* ATCC700307^T^ (marine algae) is composed of a single chromosome of 4,209,307 (bp), 3,938,396 (bp), and 4,855,835 (bp), respectively, without any plasmid.

Genome of *M. spongiae* MI-G^T^ contains a total of 4433 protein-coding sequences covering 83.36% of the genome with an average length of 842 bp. Whereas, genomes of *Microbulbifer* reference strains IRE31^T^, DAU221^T^, and ATCC700307^T^ contain 3549, 3273, and 4253 protein-coding sequences. 50 tRNAs, 12 sRNAs, 4 ncRNAs, and 4 rRNAs (5S_rRNA, 16S_rRNA, and 23S_rRNA each) were also identified in the genome of *M. spongiae* MI-G^T^. Whereas, 66, 48, and 59 tRNAs, and 4 rRNAs (5S_rRNA, 16S_rRNA, and 23S_rRNA each), 3(5S_rRNA, 16S_rRNA, and 23S_rRNA each), and 5 (5S_rRNA, 16S_rRNA, and 23S_rRNA each), were identified in the genome of three *Microbulbifer* reference strains IRE31^T^, DAU221^T^, and ATCC700307^T^.

The proportion of functional annotation of predicted coding sequences in different databases varies, as presented in Figure S3. Functional analyses of COGs, KEGGs, and GOs in the genome of four *Microbulbifer* strains showed that there are major differences in the functional categories associated with metabolism, cellular, and information in COG-functional classification (Figure S4). In addition, there are also differences in the functional categories associated with metabolism, cellular process, and environmental information processing in KEGG-functional annotation (Figure S5). GO-functional annotation also reveals differences in functional categories associated with biological process and molecular process only (Figure S6).

### 3.2. Mobile Gene Elements in Genomes of Four Microbulbifer Strains

**Figure 2:**
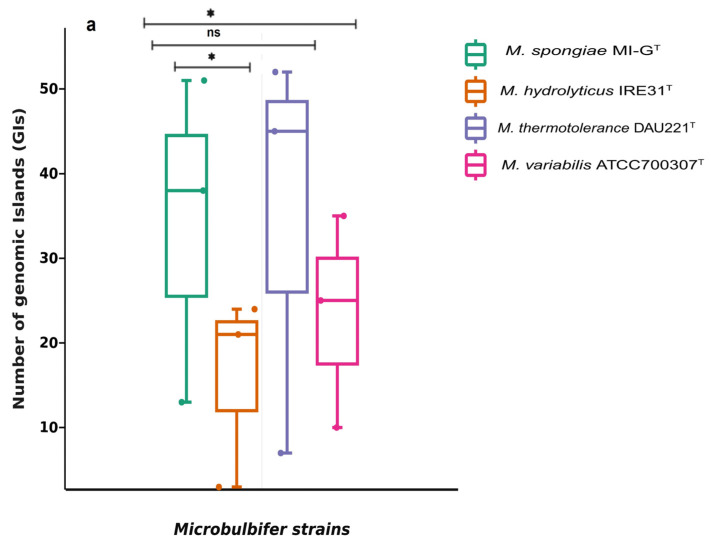

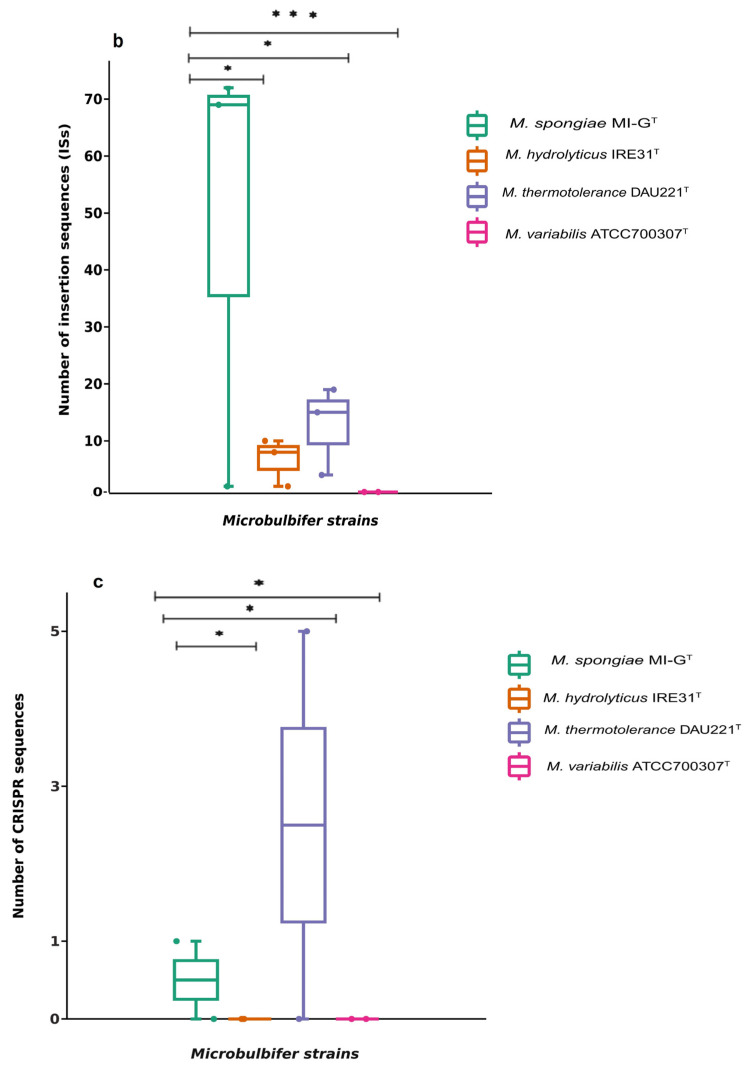
Abundance of mobile gene elements (MGEs) in the genome of sponge-associated strain *M. spongiae* MI-G^T^ and three *Microbulbifer* reference strains. Boxplots show the abundance of (**a**) genomic islands (GIs), (**b**) insertion sequences (ISs), and (**c**) CRISPR’s sequences in the genomes of *M. spongiae* MI-G^T^, *M. hydrolyticus* IRE31^T^, *M. thermotolerance* DAU221^T^, and *M. variabilis* ATCC700307^T^. Boxes represent the interquartile range (IQR), and the horizontal range line indicates the position of the median. Each dot represents one data point. The *p*-value of the statistical test on the means is indicated (* *p* < 0.05, *** *p* < 0.01, ns = non-significant).

The number of genomic islands (GIs) in the genomes of four *Microbulbifer* strains varies as 51, 24, 52, and 35 GIs in *M. spongiae* MI-G^T^, *M. hydrolyticus* IRE31^T^, *M. thermotolerance* DAU221^T^, and *M. variabilis* ATCC 700307^T^, respectively (Table S2; Figure 2a and Figure S7). A comparatively large number of insertion sequences (ISs), 72 different ISs belonging to 9 IS families, were identified in *M. spongiae* MI-G^T^. Whereas, *M. hydrolyticus* IRE31^T^ possesses 10 different ISs belonging to 7 IS families and *M. thermotolerance* DAU221^T^ has 19 different ISs belonging to 7 ISs families. In contrast, *M. variabilis* ATCC700307^T^ contains no transposable insertion element with significant similarity to ISs (Figure 2b; Table S3). Only one clustered regularly interspaced short palindromic repeat (CRISPR) sequence with two spacers was predicted in the genome of *M. spongiae* MI-G^T^. 5 CRISPR sequences with a varied number of spacers (50, 5, 4, 9, and 1) were predicted in the genome of *M. thermotolerance* DAU221^T^ (Figure 2c; Table S4). However, the other two *Microbulbifer* reference strains, i.e., *M. hydrolyticus* IRE31^T^ and *M. variabilis* ATCC700307^T^, do not have any CRISPR sequence (Figure 2c).

### 3.3. Carbohydrate Metabolism and Stress Response

**Figure 3:**
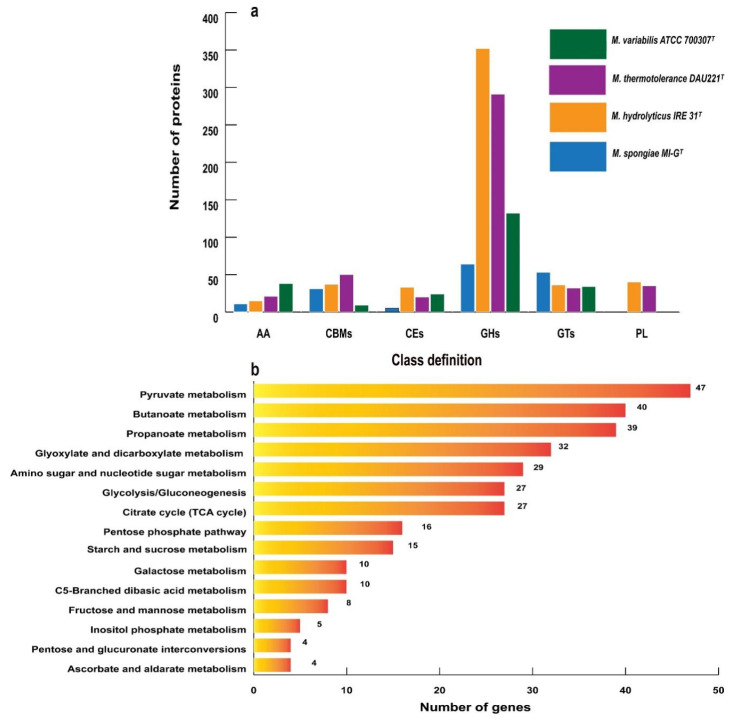
Carbohydrate metabolism of *M. spongiae* MI-G^T^ and three *Microbulbifer* reference strains. (**a**) Proportion of carbohydrate metabolism-related enzymes, annotated using the Carbohydrate-Active enzymes database. AA (auxiliary activity); CBM (carbohydrate binding module); CE (carbohydrate esterase); GH (glycoside hydrolases); GT (glycosyl transferases); PL (polysaccharide lyase) in the genomes of *M. spongiae* MI-G^T^ (blue), *M. hydrolyticus* IRE31^T^ (orange), *M. thermotolerance* DAU221^T^ (purple), and *M. variabilis* ATCC700307^T^ (green). (**b**) Abundance of genes annotated to versatile carbohydrate metabolic pathways in the genome of *M. spongiae* MI-G^T^.

The genome of *M. spongiae* MI-G^T^ was further explored by using carbohydrate-active enzyme (CAZyme) database. *M. spongiae* MI-G^T^ contains 164 CAZyme enzymes belonging to five classes of CAZymes as follows: 11 auxiliary activities (AAs), 31 carbohydrate-binding modules (CBMs), 5 carbohydrate esterases (CEs), 64 glycoside hydrolases (GHs), and 53 glycosyltransferases (GTs). We did not find any polysaccharide lyases (PLs) in the genome of *M. spongiae* MI-G^T^. However, three *Microbulbifer* reference strains (i.e., *M. hydrolyticus* IRE31^T^, *M. thermotolerance* DAU221^T^, and *M. variabilis* ATCC700307^T^) possess a comparatively higher number of CAZymes, i.e., 513, 449, and 239, respectively, than *M. spongiae* MI-G^T^. *M. hydrolyticus* IRE31^T^ possesses the highest number of CAZymes, and the number of AA, CBMs, CEs, GHs, GTs, and PL is 15, 37, 33, 352, 36, and 40, respectively. Number of AA, CBMs, CEs, GHs, GTs, and PL CAZyme-classes in *M. thermotolerance* DAU221^T^ is 21, 50, 20, 291, 32, and 35, respectively. Moreover, in *M. variabilis* ATCC700307^T^, the number of these enzymes is 38, 9, 24, 132, 34, and 2, respectively (Figure 3a). A detailed description of functional CAZyme classes and associated enzymes in the genome of *M. spongiae* MI-G^T^ and three *Microbulbifer* reference strains is listed in Table S5. These enzymes were annotated to versatile carbohydrate metabolic pathways (Figure 3b): genes associated with pyruvate metabolism, butanoate metabolism, propanoate metabolism, glyoxylate and dicarboxylate metabolism, amino sugar and nucleotide sugar metabolism, citrate cycle (TCA), and glycolysis or gluconeogenesis were more abundant than those involved in other types of carbohydrate metabolic pathways.

**Figure 4:**
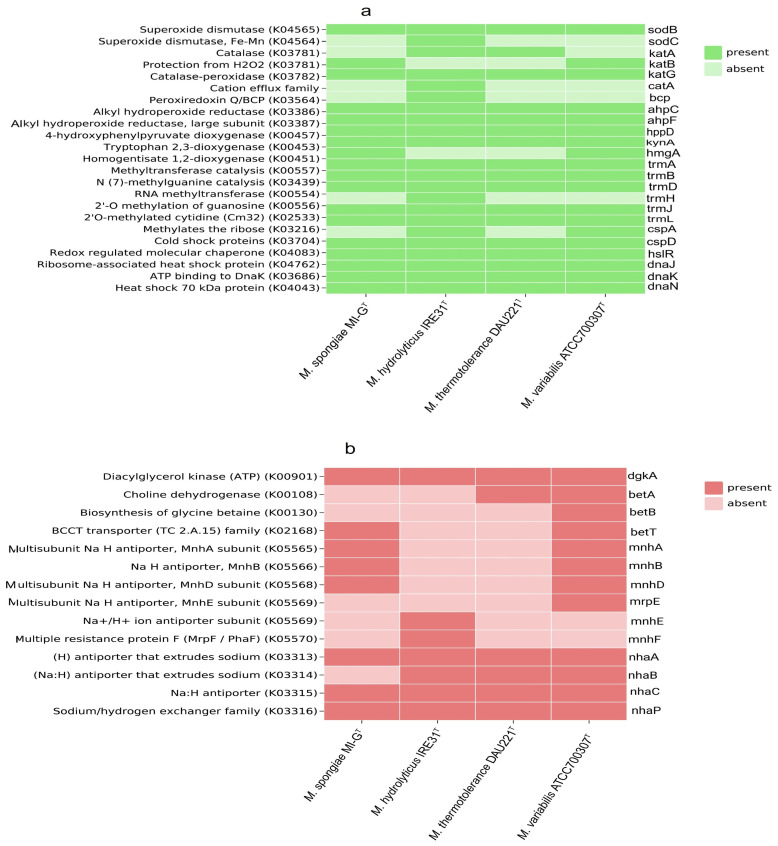

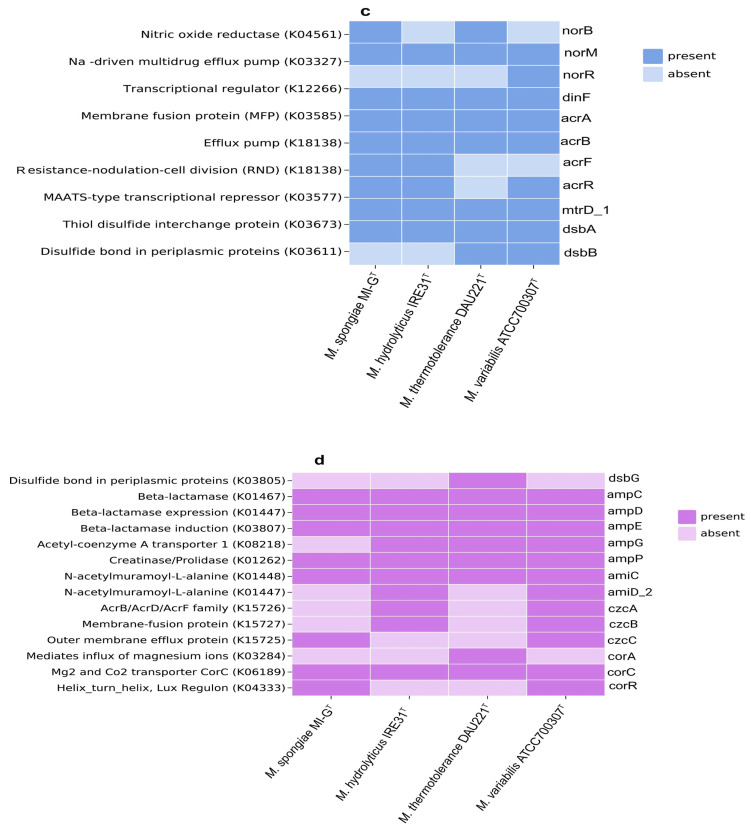
Heatmap displays the comparison of stress response proteins among sponge-associated strain *M. spongiae* MI-G^T^ and three *Microbulbifer* reference strains *M. hydrolyticus* IRE31^T^, *M. thermotolerance* DAU21^T^, and *M. variabilis* ATCC700307^T^. The color scale ranges from light green, light red, light blue, and light purple (absent) to dark green, dark red, dark blue, and dark purple, respectively (present). (**a**) Oxidative stress response in *M. spongiae* MI-G^T^ and three *Microbulbifer* reference strains. (**b**) Osmotic stress response in *M. spongiae* MI-G^T^ and three *Microbulbifer* reference strains. (**c**) Resistance to antimicrobial drugs in *M. spongiae* MI-G^T^ and three *Microbulbifer* reference strains. (**d**) Tolerance to biotoxic metals in *M. spongiae* MI-G^T^ and three *Microbulbifer* reference strains.

Genes associated with stress-response, such as oxidative stress, osmotic stress, resistance to antimicrobial drugs, and tolerance to biotoxic metals, were identified with differential distribution in the genomes of *M. spongiae* MI-G^T^ and three *Microbulbifer* reference strains. However, proteins sodC, katA, catA, bcp, trmH, cspA (oxidative stress response), betA, betT, mnhE, mnhF, nhaB (osmotic stress response), and dsbG, ampG, amiD_2, czcA, czcB, and corA (tolerance to biotoxic metals) are absent in sponge-associated strain *M. spongiae* MI-G^T^ but present in three *Microbulbifer* reference strains, as shown in Figure 4a–d and Table S1.

### 3.4. Different Genomic Characteristics of M. spongiae MI-G^T^ from Other Microbulbifer Strains

**Figure 5:**
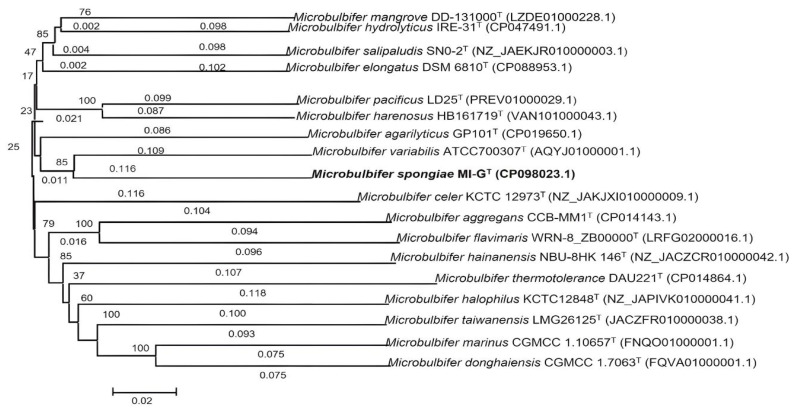
Phylogenomic tree based on genome sequences of *M. spongiae* MI-G^T^ and seventeen *Microbulbifer* reference strains using the genome blast distance phylogeny (GBDP) method. Branch lengths are scaled in terms of the GBDP distance formula d5. Numbers above branches are GBDP pseudo-bootstrap support values > 60% from 100 replications, with an average branch support of 65.4%. GenBank accession numbers are included in parentheses. Bar, 0.02 represents substitutions per nucleotide position.

Genome size of *Microbulbifer* strains including *M. spongiae* MI-G^T^, *M. hydrolyticus* IRE31^T^, *M. thermotolerance* DAU221^T^, and *M. variabilis* ATCC700307^T^ varies from 4.8 to 3.6 Mbp, and the average GC content ranges from 48.5 to 61.7%, signifying substantial inter-species variations. Phylogenomic analysis of the whole genome shows that *M. spongiae* MI-G^T^ is more closely related to *M. variabilis* ATCC700307^T^ and *M. agarilyticus* GP101^T^, indicating that *M. spongiae* MI-G^T^ is a member of the genus *Microbulbifer* (Figure 5 and Figure S8).

**Figure 6:**
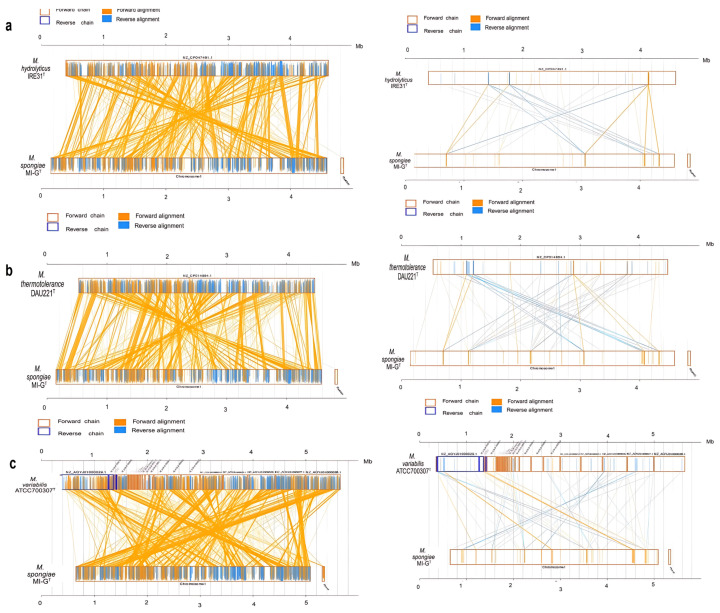
Comparative genomic analysis of *M. spongiae* MI-G^T^ with three *Microbulbifer* reference strains. (**a**) Genome-to-genome alignment of amino acid and nucleotide level sequences of *M. spongiae* MI-G^T^ to *M. hydrolyticus* IRE 31^T^. (**b**) Genome-to-genome alignment of amino acid and nucleotide level sequences of *M. spongiae* MI-G^T^ to *M. thermotolerance* DAU221^T^. (**c**) Genome-to-genome alignment of amino acid and nucleotide level sequences of *M. spongiae* MI-G^T^ to *M. variabilis* ATCC700307^T^ based on MUMmer version 3.22. In the diagram, yellow box stands for the forward chain and blue box stands for the reverse chain within the upper and following sequence region. In the box of sequence, yellow region stands for the nucleic acid sequence in forward chain of this genome sequence, and blue region stands for nucleic acid sequence in reverse chain of this genome sequence. In the middle region of two sequences, yellow line stands for forward alignment and blue line stands for reverse complementary alignment.

Furthermore, structural difference, mutation, and evolution between *M. spongiae* MI-G^T^ and three *Microbulbifer* reference strains *M. hydrolyticus* IRE31^T^, *M. thermotolerance* DAU221^T^, and *M. variabilis* ATCC700307^T^ were analyzed for genome-synteny. As shown in Figure 6, genome synteny results show high similarity, i.e., a lot of homologous regions exist in these four *Microbulbifer* strains, indicating large-scale evolutionary events have already occurred at the genus level (Figure 6a–c).

**Figure 7:**
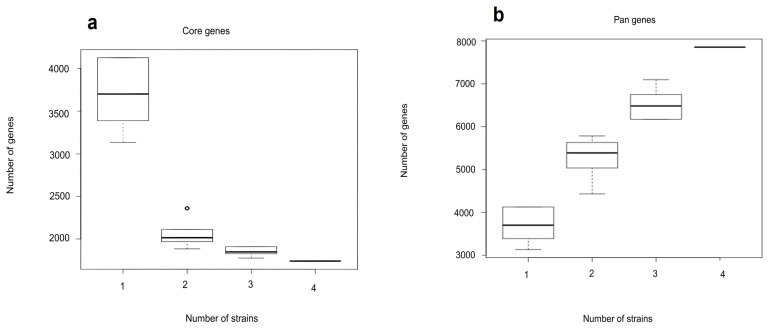
Comparative genomic analysis of *M. spongiae* MI-G^T^ with three *Microbulbifer* reference strains. (**a**,**b**) Dilution curve of core genome and pangenome.

Pangenome analysis was performed to assess the overall genetic diversity of gene repertoire in *M. spongiae* MI-G^T^ and three *Microbulbifer* reference strains. Pan-core genome analysis is important to amass molecular evidence to discriminate the diversity of genomes and to explore core, accessory, and unique genes (Figure 7a,b).

**Figure 8:**
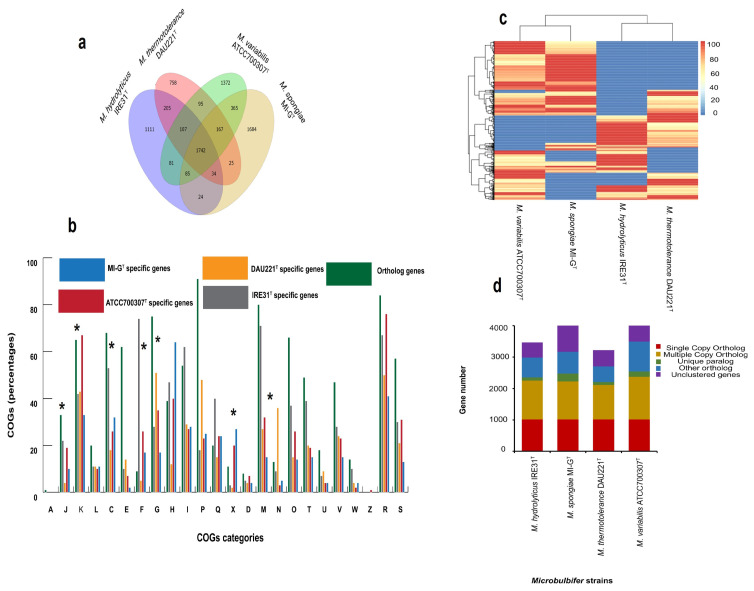
Comparative genomic analysis of *M. spongiae* MI-G^T^ with three *Microbulbifer* reference strains. (**a**) Venn diagram of orthologous, core genes, and strain-specific genes in each strain. (**b**) COG categories of orthologous and strain-specific genes in each strain. A, RNA processing and modifications; J, Translation, ribosomal structure and biogenesis; K, Transcription; L, Replication, recombination and repair; C, Energy production and conversion; E, Amino acid transport and metabolism; F, Nucleotide transport and metabolism; G, Carbohydrate transport and metabolism; H, Coenzyme transport and metabolism; I, Lipid transport and metabolism; P, Inorganic ion transport and metabolism; Q, Secondary metabolites biosynthesis, transport and metabolism; X, Mobilomes: Prophages, transposons; D, Cell cycle control, cell division, chromosome partitioning, M, Cell wall/membrane/envelope biogenesis; N, Cell motility; O, Posttranslational modification, protein turnover, chaperons; T, Signal transduction mechanisms; U, Intracellular trafficking, secretion and vesicular transport; V, Defense mechanisms; W, Extracellular structures; Z, Cytoskeleton. Asterisks indicate (*p* < 0.05 in *t*-test). (**c**) Dispensable gene heatmap of four *Microbulbifer* strains: below are each strain name, left are dispensable gene clusters, top are strain clusters, similarities of genes are shown in the middle, with different colors representing different coverage by heat map. Color/depth in top right pic. (**d**) Gene family analysis showing the orthologs among four *Microbulbifer* strains: *M. spongiae* MI-G^T^, *M. hydrolyticus* IRE31^T^, *M. variabilis* ATCC700307^T^, and *M. thermotolerance* DAU221^T^.

As shown in Figure 8c, the number of dispensable genes varies from 552 to 970, and *M. spongiae* MI-G^T^ has 773 accessory genes (19.32% of the accessory genome), *M. hydrolyticus* IRE31^T^ has 552, *M. thermotolerance* DAU221^T^ has 667, and *M. variabilis* ATCC700307^T^ has 970 accessory genes, respectively. According to the Venn graph (Figure 8d), the number of multiple-copy orthologs and un-clustered genes was much higher in sponge-associated *M. spongiae* MI-G^T^ than 3 *Microbulbifer* reference strains IRE31^T^, DAU221^T^, and ATCC 700307^T^. Venn diagram (Figure 8a) represents unique and shared family gene number: unique gene (unique orthologous cluster) family number varies from 68, 73, 47, and 39 that are specific to *Microbulbifer* strains MI-G^T^, IRE31^T^, DAU221^T^, and ATCC700307^T^, respectively. Ortholog analysis led to the identification of genes present in different orthologs in these four *Microbulbifer* strains. As shown in (Figure 8a), 1742 core conserved genes, 7855 pan genes and 1188 dispensable genes were identified in these four *Microbulbifer* strains (Figure 8a). As far as the strain-specific genome is concerned, *M. spongiae* MI-G^T^ has 1879 unique genes, whereas *M. hydrolyticus* IRE31^T^ has 1144 unique genes, *M. thermotolerance* DAU221^T^ has 784 unique genes, and *M. variabilis* ATCC 700307^T^ has 1466 unique genes; the combine total unique genes are 5273 (34.39% of the pangenome); while the number of core genes ranges from 1757 to 1789, and *M. spongiae* MI-G^T^ has 1781 core genes (46.26% of the core genome) (Figure 8a). As shown in Figure S9. *M. spongiae* MI-G^T^ has 68 unique family gene numbers, whereas *M. hydrolyticus* IRE31^T^ and *M. thermotolerance* DAU221^T^ possess comparatively lower numbers of unique family gene numbers.

Genes of four *Microbulbifer* strains were functionally classified according to COG-functional classification (Figure 8b; Table S6). Figure S9 indicates COG-functional distribution of gene families in core genome of four *Microbulbifer* strains. Core genes up to 1742 are conserved in *M. spongaie* MI-G^T^ and three *Microbulbifer* strains. Whereas, sponge-associated strain *M. spongiae* MI-G^T^ has a comparatively larger number of strain-specific genes than three *Microbulbifer* reference strains ATCC700307^T^, IRE31^T^, and DAU221^T^ (Figure 8a). According to our analysis (Table S6 and Figure 8b), there are differences in the functional distribution of genes belonging to different COG-classes in *M. spongiae* MI-G^T^ and three *Microbulbifer* reference strains IRE31^T^, DAU221^T^, and ATCC700307^T^. Most obvious difference was noted in the functional-distribution of strain-specific genes belonging to metabolism such as nucleotide transport and metabolism (F), energy production and conversion (C), carbohydrate transport and metabolism (G), coenzyme transport and metabolism (H), lipid transport and metabolism (I), amino acid transport and metabolism (E), inorganic ion transport and metabolism (P), secondary metabolite biosynthesis, transport and catabolism (Q), mobilome: prophages, transposons (X). In cellular system, significant difference was identified in the functional distribution of strain-specific genes in 4-classes i.e., cell cycle control, cell division, chromosome partitioning (D), cell motility (N), cell wall/membrane/envelope biogenesis (M), and signal transduction mechanisms (T), while less significant difference was observed in remaining 6-classes such as posttranslational modification, protein-turnover, chaperones (O), cytoskeleton (Z), function unknown (S), intracellular trafficking, secretion, and vesicular transport (U), defense mechanisms (V) and extracellular structures (W). In information storage and processing system, most pronounced difference in functional distribution of strain-specific genes was observed in COG-classes such as transcription (K), translation, ribosomal structure and biogenesis (J), whereas, in RNA processing and modification (A), replication, recombination and repair (L), difference was decreased in four *Microbulbifer* strains. Moreover, strain-specific genes associated with category translation, ribosomal structure and biogenesis, transcription, nucleotide transport and metabolism, energy production and conversion, carbohydrate transport and metabolism, and cell motility are lower in sponge-associated strain *M. spongiae* MI-G^T^ compared to strain-specific genes of three *Microbulbifer* reference strains (*p* < 0.05). However, strain-specific genes associated with mobilome, i.e., prophages, transposons, are exceptionally higher in *M. spongiae* MI-G^T^, compared to *Microbulbifer* reference strains (*p* < 0.05) (Table S6).

### 3.5. Sponge-Bacteria Metabolic Interaction Indicated by Genome Analysis

**Figure 9:**
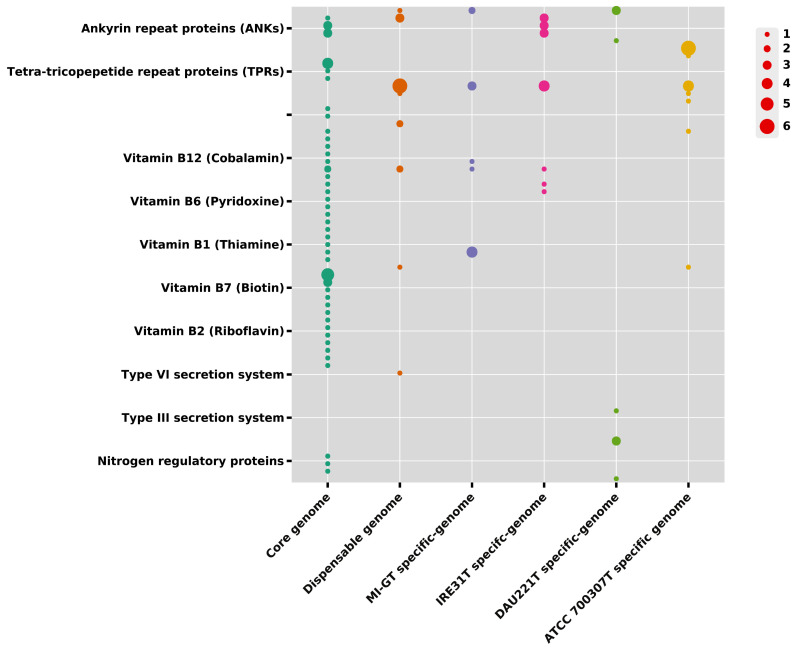
Bubble plots show the difference in abundance and statistical significance of specific COGs related to metabolic interaction-related features in core genome (green color), dispensable genome (orange color), *M. spongiae* MI-G^T^ (purple color), and three *Microbulbifer* reference strains *M. hydrolyticus* IRE31^T^ (pink color), *M. variabilis* ATCC700307^T^ (yellow color), and *M. thermotolerance* DAU221^T^ (light-green color). The size of the circle indicates statistically significant difference (*p* < 0.05, in *t*-test), with larger circles representing a greater number of counts per COGs, representing smaller *p*-values and thus more pronounced difference.

Eukaryotic-like proteins (ELPs), such as ankyrin-repeat proteins (ANKs) and tetra-tricopepetide repeat proteins (TPRs), are mostly used for the establishment of host-microbe association in the holobiont concept [59,60,61]. According to this study, we identified one ANK (COG0666) protein in *M. spongiae* MI-G^T^ and *M. thermotolerance* DAU221^T^ (Figure 9). *M. spongiae* MI-G^T^ has 2 members, while free-living *M. thermotolerance* DAU221^T^ has 3 members of the ANK (COG0666) protein. The other two *Microbulbifer* reference strains, *M. hydrolyticus* IRE31^T^ and *M. variabilis* ATCC700307^T^, do not possess ankyrin repeat proteins. Tetra-tricopepetide repeat proteins (TPRs) were identified with differential distribution in four *Microbulbifer* strains. 3 members of each TPR protein (COG4783, COG0457, and COG3118) were identified in *M. hydrolyticus* IRE31^T^, while only one member of TPR protein (COG4941) was identified in free-living *M. thermotolerance* DAU221^T^. Moreover, 6 and 1 members of TPR proteins (COG4976 and COG4700) were observed only in *M. variabilis* ATCC700307^T^. However, we did not identify any TPR protein specific to *M. spongiae* MI-G^T^. It seems that most of the TPR proteins may be conserved in 4 *Microbulbifer* strains. According to genome analysis, vitamin B12 (cobalamin), vitamin B6 (pyridoxine), vitamin B1 (thiamine), vitamin B7 (biotin), and vitamin B2 (riboflavin) were identified with different numbers of repeats per vitamin in *M. spongiae* MI-G^T^, *M. hydrolyticus* IRE31^T^, *M. thermotolerance* DAU221^T^, and *M. variabilis* ATCC700307^T^. Fourteen type VI secretion system proteins were detected, and no type-III secretion system protein was identified (Table S7; Figure 9). Ten type-III secretion system proteins were observed only in the free-living strain *M. thermotolerance* DAU221^T^, and no protein of this system was present in *M. spongiae* MI-G^T^, *M. hydrolyticus* IRE31^T^, and *M. variabilis* ATCC700307^T^ (Table S7).

## 4. Discussion

In this study, we investigate the genomic diversity, metabolic characteristics, and functional evolution of sponge-associated strain *M. spongiae* MI-G^T^ by performing whole-genome sequencing and comparative genomic analysis. The genome size of *M. spongiae* MI-G^T^ (4,478,124 bp) is slightly larger than the genome sizes of free-living strains *M. hydrolyticus* IRE31^T^ (4,209,307 bp) and *M. thermotolerance* DAU221^T^ (3,938,396 bp) (Table 1). Moreover, *M. spongiae* MI-G^T^ had a smaller GC content than free-living strains IRE31^T^ and DAU221^T^. Reduced genome size and low GC content are the main characteristics of obligate intracellular symbionts [62]. Our results indicate that sponge-associated *M. spongiae* MI-G^T^ is probably a facultative sponge-symbiont [63]. *M. spongiae* MI-G^T^ may have a free-living stage and therefore retain the necessary genes for both free-living and host-associated lifestyles. A higher number of pseudogenes in the genome of *M. spongiae* MI-G^T^ suggests that this bacterium may possibly have undergone a process of pseudogenization as an adaptation to its host environment. This evolutionary strategy highlights how microorganisms can rapidly adjust to specific niches through genomic changes, and understanding such adaptations contributes to our knowledge of the evolution of new bacterial species [64]. This suggestion is supported by the finding that these *Microbulbifer* strains occupy very divergent lineages (Figure 5), and this diversity provides important clues about their adaptation to different environments [24,65]. Meanwhile, a variety of proteins related to oxidative stress, osmotic stress, resistance to antimicrobial drugs, and tolerance to biotoxic metals were identified in the genomes of *M. spongiae* MI-G^T^ and three *Microbulbifer* reference strains (Table S1; Figure 4). Most of the proteins related to these stress response factors are conserved in four *Microbulbifer* strains. The identification of eukaryotic-like proteins (AR and TPR), vitamins, and type VI secretion system proteins in sponge-associated *M. spongiae* MI-G^T^ and three *Microbulbifer* reference strains suggests that genetic material may have been transferred between different species of *Microbulbifer* bacteria, leading to the acquisition of new genetic traits [61,66,67]. This indicates the dynamic nature of bacterial evolution and adaptation to different environments through genetic exchange mechanisms [61,66,67].

Functional genomic analysis reveals that the *M. spongiae* MI-G^T^ genome is enriched in genes and metabolic pathways for carbohydrate metabolism and stress response (Tables S1 and S5). However, *M. spongiae* MI-G^T^ possesses the least number of CAZymes when compared to three *Microbulbifer* reference strains (Figure 3a). Among the five CAZyme classes that were observed in the genome of *M. spongiae* MI-G^T^, GHs (glycoside hydrolases) were predominant over the other types of CAZymes, and the PL (polysaccharide lyases) were the least abundant. This supports previous findings that a higher number of GHs compared to other classes of CAZymes were found in the sponge microbiomes [68,69]. GHs have been classified into various families based on their amino acid sequences and structural similarities, as shown in Table S5. GHs are generally involved in the hydrolysis of glycosidic linkages between monosaccharides, which can lead to the release of simple sugars and oligosaccharides. Polysaccharide lyases (PLs) degrade polysaccharides by cleaving glycosidic bonds through an elimination mechanism rather than hydrolysis. PLs are usually involved in the breakdown of various complex carbohydrates such as pectin’s, and alginates, facilitating their utilization by microbes and plants [70]. The absence of PLs in *M. spongiae* MI-G^T^ suggests that sponge-associated bacteria may not require PLs for carbohydrate breakdown, possibly because their nutrient source differs from those of free-living or other host-associated bacteria [71,72]. *M. spongiae* MI-G^T^ exhibits a higher number of genes associated with pyruvate metabolism, butanoate metabolism, propanoate metabolism, glyoxylate and dicarboxylate metabolism, amino sugar and nucleotide sugar metabolism, and citrate cycle (TCA) pathways compared to other carbohydrate metabolic pathways (Figure 3b). Genes encoding proteins required for major reactions in the glycolysis and pentose phosphate pathway, the tricarboxylic acid (TCA) cycle and oxidative phosphorylation have been identified in the genomes of sponge-associated microorganisms [73,74], whereas, in *M. spongiae* MI-G^T^, the number of these genes are lower compared to genes associated with other pathways such as pyruvate metabolism, butanoate metabolism, propanoate metabolism, glyoxylate and dicarboxylate metabolism, suggesting that *M. spongiae* MI-G^T^ may use a wide range of sugar and carbon compounds to meet its nutritional needs [10].

It is known that stress-related genes play a significant role in the survival of bacterial species under stressed environmental conditions [75]. However, several proteins sodC, katA, catA, bcp, trmH, cspA (oxidative stress response), betA, betT, mnhE, mnhF, nhaB (osmotic stress response), and dsbG, ampG, amiD_2, czcA, czcB, and corA (tolerance to biotoxic metals) (Figure 4a–d), are absent in *M. spongiae* MI-G^T^ but present in three *Microbulbifer* reference strains, suggesting that sponge-associated strain *M. spongiae* MI-G^T^ may be more tolerant to environmental stress than *Microbulbifer* reference strains [76]. Moreover, the absence of these proteins might indicate their evolutionary divergence from *Microbulbifer* reference strains, because the sponge host may provide a stable environment for *M. spongiae* MI-G^T^ that mitigates the need for such stress response-related factors [77]. For example, periplasmic sodC (superoxide dismutase) is absent in *M. spongiae* MI-G^T^, only identified in the free-living strain *M. hydrolyticus* IRE31^T^. SodC is a Cu/Zn periplasmic superoxide dismutase protein that is produced by bacteria to combat toxic superoxide radicals to H_2_O_2_ to O_2_ through the alternate oxidation and reduction of the copper ion in the active site. This suggests that sponge-associated *M. spongiae* MI-G^T^ residing in the sponge host environment may not encounter toxic superoxide radicals [63]. Similarly, catalase genes katA and catA that are required for hydrogen peroxide (H_2_O_2_) resistance are also absent in *M. spongiae* MI-G^T^, but present in free-living strains *M. hydrolyticus* IRE31^T^ and *M. thermotolerance* DAU221^T^, suggesting that free-living *Microbulbifer* strains may encounter more reactive oxygen species (ROS) than the sponge-associated strain *M. spongiae* MI-G^T^ [78,79]. Moreover, glycine, betaine, and choline genes *betA*, *betB*, *betT,* and Na^+^ pump associated genes *mrpE*, mnhE, *mnhF* and *nhaB* that are involved in the response to osmotic stress are also absent in *M. spongiae* MI-G^T^, but present in three *Microbulbifer* reference strains, indicating that sponge-associated strain *M. spongiae* MI-G^T^ occupies a unique niche where osmotic stress is not a significant challenge [80].

Functional analysis of COGs, KEGGs, and GOs in the genomes of four *Microbulbifer* strains reveals significant variations in their cellular and informational functions, which may result from various evolutionary pressures such as nutrient availability, environmental conditions, and interactions with their host [81]. It is known that the interplay between genetic drift, mutational bias towards deletion, accumulation of pseudogenes, and mobile gene elements plays a pivotal role in shaping bacterial genome size and gene content. These factors are able to influence bacterial evolution, genetic diversity, and genomic characteristics of bacterial species [82,83,84,85,86]. In this study, only one plasmid was identified in the *M. spongiae* MI-G^T^ strain, whereas three *Microbulbifer* reference strains do not have any plasmids. Plasmids are genetic elements for colonization and replication, and they are believed to be a major driving force of bacterial evolution as they can migrate between populations to induce lateral DNA transfer [81]. Oliveira et al. highlight the importance of plasmids in transferring genetic traits putatively involved in bacterial symbiont adaptation and sponge-bacteria interaction [87]. Horizontal plasmid transfer events in the sponge microbiome may enable the symbiotic bacteria to recruit a range of accessory genes relevant to signaling, stress-responsive factors, and production of amino acids that may contribute to the establishment of a mutualistic relationship between the sponge and bacteria [88,89,90].

Higher number of mobile gene elements (MGEs) have already been reported in the genome of sponge-associated bacteria and plays a critical role in their evolution and metabolic interaction with the host organisms [18,73,91]. It seems that genetic transfer by MGEs in sponge-associated strain *M. spongiae* MI-G^T^ is an indispensable phenomenon for this bacterium to adapt sponge-host environment [81]. Genomic islands (GIs) are typically acquired through horizontal gene transfer (HGT) and play a role in enhancing the survival of bacteria in diverse environments [92]. GIs are important components of bacterial genomes that contribute significantly to their evolution and adaptation [81]. A higher number of GIs detected in *M. spongiae* MI-G^T^ (Figure 4a) indicates the possible role of GIs in its evolution in order to adapt to its sponge host environment. Meanwhile, *M. spongiae* MI-G^T^ possesses a comparatively higher number of insertion sequence (IS) elements (72 belonging to eleven IS families) than three *Microbulbifer* reference strains (1–19) (Figure 4b). Our results are consistent with previous findings that sponge-associated bacteria contain almost three to five times of ISs elements compared to free-living or other host-associated bacteria [18]. Certain IS families, such as IS256 and IS66, have been found to be exclusively associated with sponge-associated bacteria [93]. IS256 is also a significant element in *Enterococcus faecalis* and *E. faecium*, closely linked to their antimicrobial resistance traits [94]. IS66 is an important IS element characterized by three ORFs (i.e., a transposase and two regulatory genes), influencing the genetic landscape and contributing to resistance to environmental stress [95]. The presence of a higher number of ISs in sponge-associated strain *M. spongiae* MI-G^T^ may be linked to its adaptation to the sponge-host, as their transposition can result in gene inactivation and modulation of surrounding gene expression. Such genetic variability may enhance the bacteria’s capacity for survival and interaction within the sponge-host environment [96]. In addition to GIs and IS, clustered regularly interspaced short palindromic repeats (CRISPRs) were also identified only in *M. spongiae* MI-G^T^ and *M. thermotolerance* DAU221^T^, while the other two strains do not possess any CRISPR sequences. CRISPR-Cas’s system is a type of adaptive immunity in bacteria, which protects them against invading genetic elements [97]. The presence of CRISPR-Cas system in *M. spongiae* MI-G^T^ suggests that this bacterium could trigger defence mechanisms against the invasion of exogenous DNA for maintaining the stability of genetic architecture [98]. Interestingly, the number of unique paralogs is quite higher in *M. spongiae* MI-G^T^ compared to the three *Microbulbifer* reference strains (Figure 8d). A paralog is a type of gene that arises through a gene duplication event within a genome. After duplication, these genes can evolve independently, leading to the potential acquisition of new functions within organisms. Unique paralog genes are critical in the evolutionary process, influencing both genetic diversity and emergence of new species [99]. Similarly, the number of dispensable genes is also higher in *M. spongiae* MI-G^T^ than in free-living strains *M. thermotolerance* DAU221^T^ and *M. hydrolyticus* IRE31^T^. Tong et al. found that dispensable genes that appear to be non-functional under normal environmental conditions become crucial when bacteria face stressful situations [100]. It can be suggested that *M. spongiae* MI-G^T^ may use dispensable genes to combat environmental stress in a challenging marine environment. Moreover, the presence of a few numbers of genes related to cell motility in *M. sponge* MI-G^T^ compared to three *Microbulbifer* reference strains also indicates a sponge-associated lifestyle of *M. spongiae* MI-G^T^. The loss of motility genes in sponge-associated bacteria has previously been reported and hypothesized to be a cause of a mutualistic lifestyle and vertical transmission of symbiotic bacteria [101,102].

Sponge-associated strain *M. spongiae* MI-G^T^ possesses a higher number of strain-specific genes and unique family number genes compared to three *Microbulbifer* reference strains (Figure 8a and Figure S9a), which is consistent with previous findings that sponge-associated bacteria possess a comparatively higher number of strain-specific genes than other strains of the same genus isolated from other habitats [18,103]. There are differences in the functional distribution of genes belonging to different COG-categories in four *Microbulbifer* strains (Table S5; Figure 8b). Strain-specific genes associated with functional-category translation, ribosomal structure and biogenesis, transcription, nucleotide transport and metabolism, energy production and conversion, carbohydrate transport and metabolism, and cell motility are lower compared to those of three *Microbulbifer* reference strains. It is known that sponge-associated bacteria are engaged in nutritional exchange with their sponge-hosts [1,104], and have evolved metabolic dependencies on their sponge-hosts [27]. Thus, it can be hypothesized that sponge-associated strain *M. spongiae* MI-G^T^ may acquire nutrients and energy from its sponge host thus possess a lower number of strain-specific genes related to metabolism and energy production compared to free-living strains.

**Figure 10:**
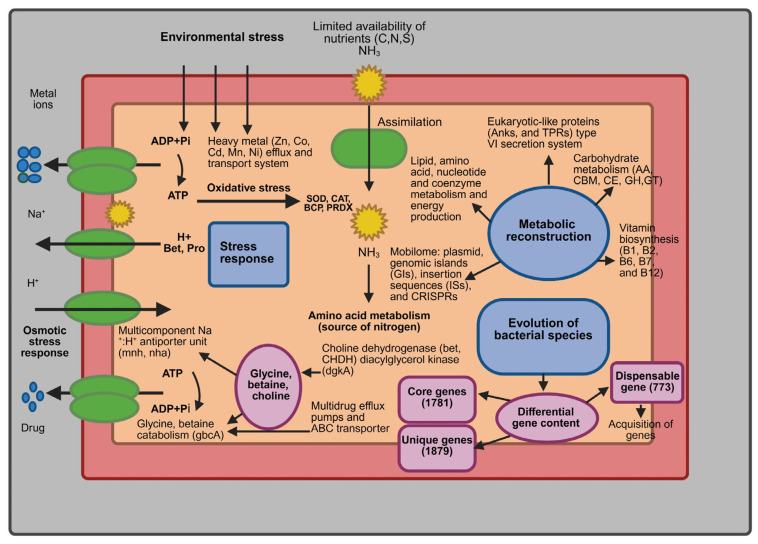
Schematic overview of genomic characteristics that reflect evolutionary adaptation of *M. spongiae* MI-G^T^.

The genome and comparative genomic analysis of *M. spongiae* MI-G^T^ with three *Microbulbifer* reference strains provide unique insights into the genomic repertoire of a given phylogenetic taxon and functional metabolic distribution. As summarized in Figure 10, sponge-associated strain *M. spongiae* MI-G^T^ exhibits gene characteristics related to its adaptation to the sponge host habitat. Genomic strategies that enable *M. spongiae* MI-G^T^ to adapt a sponge-host associated lifestyle include: the combined role of MGE-mediated gene transfer and reduction in metabolic, energy production, motility, certain stress responsive-related genes, and the acquisition of unique strain-specific genes in *M. spongiae* MI-G^T^ offer new insights into bacterial survival in the sponge-host environment.

Previous analysis of sponge-associated bacteria is also based on metagenomic binning [105,106]. In this study, all the findings are based on genomic sequencing data, which makes our results sound robust than metagenomic binning data. However, the genome-based analyses are theoretical and require experimental validation to confirm the functional roles of identified genes or proteins. In addition, functional genomic characterization of sponge-associated bacteria *M. spongiae* MI-G^T^ presents several inherent limitations that can affect the accuracy and comprehensiveness of our findings, because the interpretation of genomic data heavily relies on existing databases and annotation tools, which may not include novel genes or functions unique to *M. spongiae* MI-G^T^. Thus, the major limitation is that genome sequences may contain unannotated regions or misannotations, leading to gaps in understanding the full metabolic and functional genomic characteristics of *M. spongiae* MI-G^T^. Moreover, the suggestions of the functional genomic characteristics based on genomic analysis need to be further elucidated in the near future through genetic expression, gene knockout, and specific protein characterization experiments.

## 5. Conclusions

The genus *Microbulbifer* is an exemplary group of marine bacteria that showcases remarkable adaptability to diverse environments. This study investigates the genomic diversity, functional evolution, and metabolic characteristics of *M. spongiae* MI-G^T^ by employing comprehensive genomic and comparative genomic approaches. Large genome size and lower GC content suggest that *M. spongiae* MI-G^T^ may be a facultative sponge-symbiont. Comparative genomic analysis indicates that there are differences in the functional distribution of genes belonging to different COG-classes among four *Microbulbifer* strains. We further identified that *M. spongiae* MI-G^T^ possesses a higher number of strain-specific genes than reference strains, suggesting that acquisition of unique genes is critical for this bacterium to adapt sponge-host environment. Moreover, strain-specific genes related to metabolism and energy production are significantly lower in *M. spongiae* MI-G^T^, suggesting that this bacterium may have evolved metabolic dependency on its sponge-host. The loss of motility genes in *M. spongiae* MI-G^T^ is also a characteristic of a mutualistic lifestyle or sponge-associated lifestyle. Interestingly, superoxide dismutase, catalase, glycine, betaine, and choline, Na^+^ pump, and tolerance to biotoxic metal proteins related genes are absent in *M. spongiae* MI-G^T^ but present in three reference *Microbulbifer* strains, indicating that the sponge-associated lifestyle of *M. spongiae* MI-G^T^ enables this bacterium to withstand environmental stress more effectively than reference *Microbulbifer* strains. Finally, the significantly larger abundance of mobile genetic elements (MGE) is the most distinctive feature between sponge-associated strain *M. spongiae* MI-G^T^ and three *Microbulbifer* reference strains. This could be linked to the sponge-associated lifestyle of *M. spongiae* MI-G^T^. Our results suggest a variety of ways *M. spongiae* MI-G^T^ have adapted to survive in the sponge-host environment. Although key sponge-host-associated traits exist in *M. spongiae* MI-G^T^, our analysis suggests that this bacterium also carries its own unique characteristics that reflect its evolution towards a sponge-associated lifestyle.

## Figures and Tables

**Table 1:** General genome features of *M. spongiae* MI-G^T^, and three *Microbulbifer* reference strains.

Category	MI-G^T^	IRE31^T^	DAU221^T^	ATCC700307^T^
Genome size (nt)	4,478,124 (bp)	4,209,307 (bp)	3,938,396 (bp)	4,855,835 (bp)
Plasmid	1	N/A	N/A	N/A
GC content (%)	54.51	57.6	56.6	49.5
Protein-coding genes	4433	3549	3273	4253
Genes with functions	3683	3440	3145	4142
Contig Number	2	1	1	1
Completeness (%)	100	100	99.44	99.44
Contamination (%)	0.56	0.56	0	0.56
ncRNA	4	4	4	4
tRNA	50	66	48	59
5s_rRNA	4	4	3	5
16s_rRNA	4	4	3	5
23s_rRNA	4	4	3	5
Pseudogenes	83	27	67	33
Genome coverage	267.0x	400.0x	197.8x	100x

Note: *M. spongiae* MI-G^T^ (this study) (CP098023.1; Chromosome), *M. spongiae* MI-G^T^ (this study) (CP098024.1; Plasmid), *M. hydrolyticus* IRE31^T^ (CP047491.1), *M. thermotolerance* DAU221^T^ (CP014864.1), *M. variabilis* ATCC700307^T^ (CP092418.1). ‘T’ superscript indicates that the bacterial strain is a type-strain.

## Data Availability

The GenBank (https://www.ncbi.nlm.nih.gov/datasets/genome/GCF_030440425.1, accessed on 31 May 2022) accession number of the whole-genome sequence of *M. spongiae* MI-G^T^ is CP098023.1, CP098024.1 for the chromosome and plasmid, respectively.

## Supplementary Materials

The following supporting information can be downloaded at: https://www.mdpi.com/article/10.3390/microorganisms13081940/s1. Figure S1: *M. spongiae* MI-G^T^ DNA purity and integrity were assessed by 0.5% agarose gel electrophoresis. Figure S2: Workflow of the pipeline outlines the steps involved in processing, annotating, and analyzing the genome of *M. spongiae* MI-G^T^. Figure S3: Proportions of genes annotated in nine databases; Figure S4: COG functional distribution of gene families in genome of *M. spongiae* MI-G^T^, and three reference *Microbulbifer* strains *M. hydrolyticus* IRE31^T^, *M. thermotolerance* DAU221^T^, and *M. variabilis* ATCC700307^T^; Figure S5: KEGG functional distribution of gene families in genome of *M. spongiae* MI-G^T^, and three *Microbulbifer* reference strains *M. hydrolyticus* IRE31^T^, *M. thermotolerance* DAU221^T^, and *M. variabilis* ATCC700307^T^; Figure S6: GO functional distribution of gene families in genome of *M. spongiae* MI-G^T^, and three *Microbulbifer* reference strains *M. hydrolyticus* IRE31^T^, *M. thermotolerance* DAU221^T^, and *M. variabilis* ATCC700307^T^; Figure S7: Computational identification and visualization of genomic islands (GIs) in (a) *M. spongiae* MI-G^T^ and three *Microbulbifer* reference strains (b) *M. hydrolyticus* IRE31^T^, (c) *M. thermotolerance* DAU221^T^, and (d) *M. variabilis* ATCC700307^T^; Figure S8: Neighbor-joining (NL) tree based on single-copy orthologous protein sequences of *M. spongiae* MI-G^T^ and three *Microbulbifer* reference strains. Amino acid sequences were identified using Proteinortho version 6.0 software with cutoff criteria of e-value 1e-5, sequence identity 50%, and sequence coverage 50%. The NL tree was calculated with 1000 bootstrap replicates. Bar, 0.02 represents substitution per amino acid sequence; Figure S9: Comparative analysis of *M. spongiae* MI-G^T^ with three reference *Microbulbifer* strains (*M. hydrolyticus* IRE31^T^, *M. thermotolerance* DAU221^T^, and *M. variabilis* ATCC700307^T^). (a) Venn diagram showing shared and unique gene family numbers among *Microbulbifer* strains: *M. spongiae* MI-G^T^, *M. hydrolyticus* IRE-31^T^, *M. variabilis* ATCC700307^T^, and *M. thermotolerance* DAU221^T^. (b) COG functional distribution of gene families in the core genome of four *Microbulbifer* strains; Table S1: Stress-responsive genes and their respective annotations in the genome of *M. spongiae* MI-G^T^ and three *Microbulbifer* reference strains; Table S2: Genomic Island (GIs) statistics in *M. spongiae* MI-G^T^ and three *Microbulbifer* reference strains; Table S3: Insertion sequence (ISs) element statistics in *M. spongiae* MI-G^T^ and three *Microbulbifer* reference strains; Table S4: Predictive statistics and description of CRISPR in *M. spongiae* MI-G^T^ and three *Microbulbifer* reference strains; Table S5. Classification of putative carbohydrate-Active enzymes, corresponding coding genes and (annotation), present in the genome of *M. spongiae* MI-G^T^ and three *Microbulbifer* reference strains; Table S6: Comparison of COG functional classes of *M. spongiae* MI-G^T^ with three *Microbulbifer* reference strains; Table S7: Comparison of metabolic features of *M. spongiae* MI-G^T^ with three *Microbulbifer* reference strains.
