# Supplementary material for: Functional Genomic Characteristics of Marine Sponge-Associated Microbulbifer spongiae MI-GT
Source: Microorganisms. 2025 Aug 20;13(8):1940. doi: 10.3390/microorganisms13081940 (PMC12388028; doi:10.3390/microorganisms13081940)
Supplement: Supplementary file 1 [file microorganisms-13-01940-s001.zip › microorganisms-3755873-supplementary.pdf]

Supplementary material

Functional genomic characteristics of marine sponge-associated *Microbulbifer spongiae* MI-G^T

Nabila Ishaq[1], Qianqian Song[1], Micha Ilan[4], Zhiyong Li[1,2,3*]

[1]Marine Biotechnology Laboratory, State Key Laboratory of Microbial Metabolism and School of Life Sciences and Biotechnology, Shanghai Jiao Tong University, Shanghai 200240, P.R. China

[2]Hainan Research Institute, Shanghai Jiao Tong University, Sanya 572025, P.R. China

[3]Joint International Research Laboratory of Metabolic & Developmental Sciences, Shanghai Jiao Tong University, Shanghai 200240, China

[4]School of Zoology, Faculty of Life Sciences, Tel- Aviv University, Tel-Aviv, Israel

# Correspondence to Zhiyong Li, zyli@sjtu.edu.cn

Data on DNA concentration, DNA integrity, and purity

M. spongiae MI-G^T DNA concentration: 225.25 ng/ul

A260/A280 2.022

A260/A230 2.366

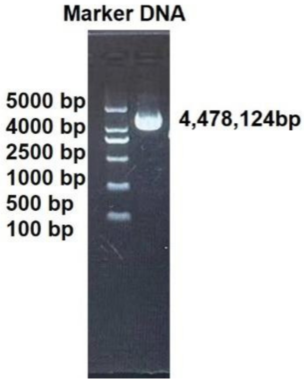

Figure S1. *M. spongiae* MI-G^T DNA purity and integrity was assessed by 0.5 % agarose gel electrophoresis.

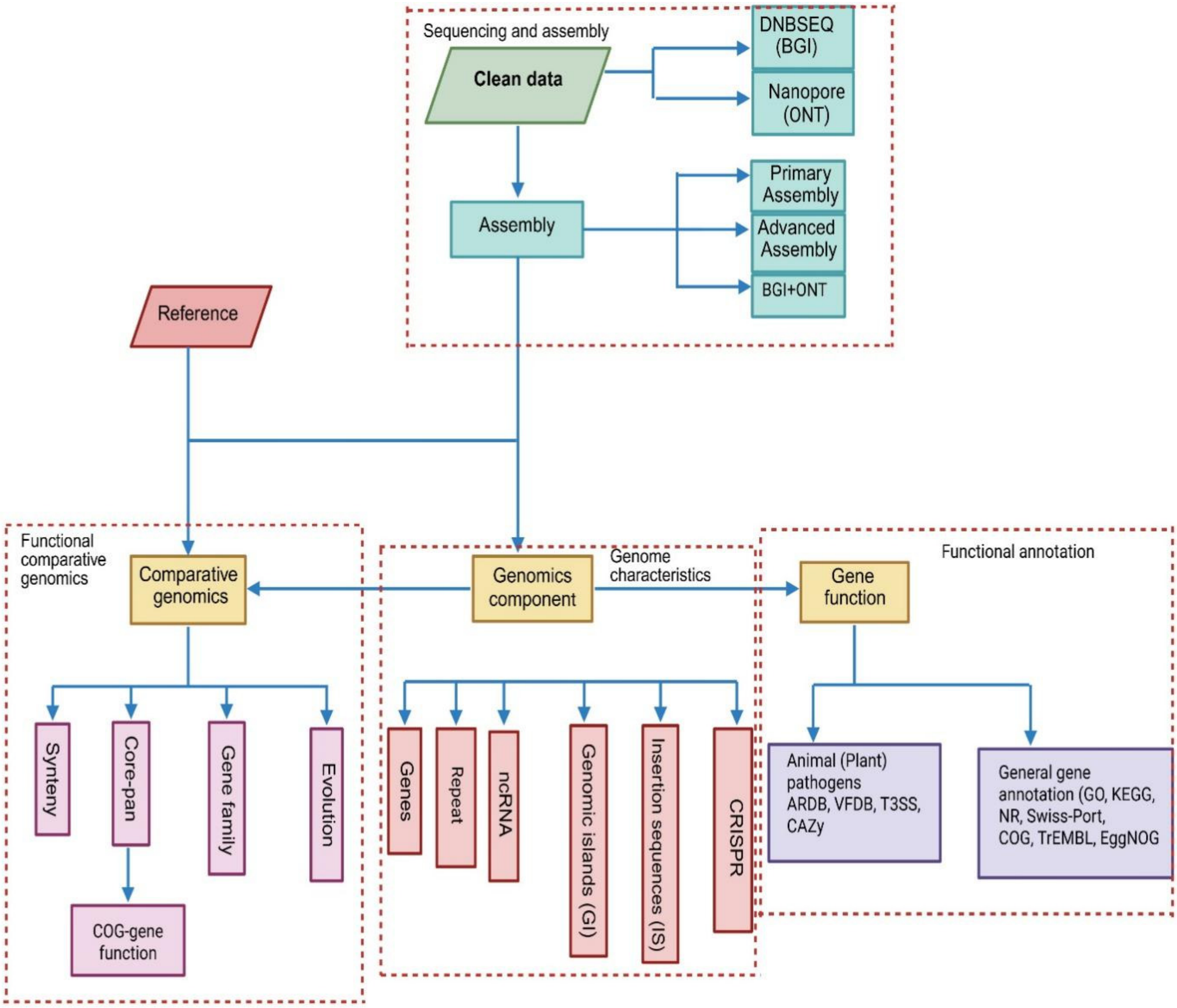

Figure S2. Workflow of the pipeline outlines the steps involved in processing, annotating and analyzing the genome of *M. spongiae* MI-G^{T}

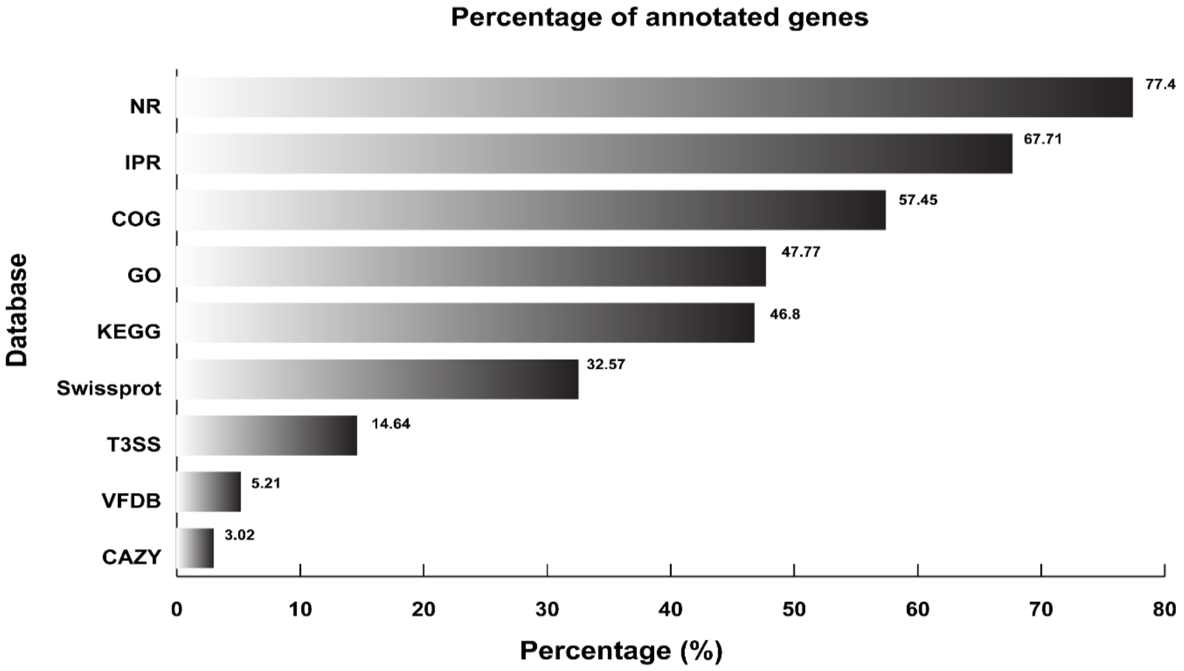

Figure S3. Proportions of genes annotated in nine databases

NR - Non-Redundant

IPR- Integrated Plant Record

COG- Clusters of Orthologous Genes

GO-Gene Ontology

KEGG-Kyoto Encyclopedia of Genes and Genomes

SWISS-PROT- Swissprot

T3SS-Type III Secretion System

VFDB-Virulence factor database

CAZY-Carbohydrate-active enzyme

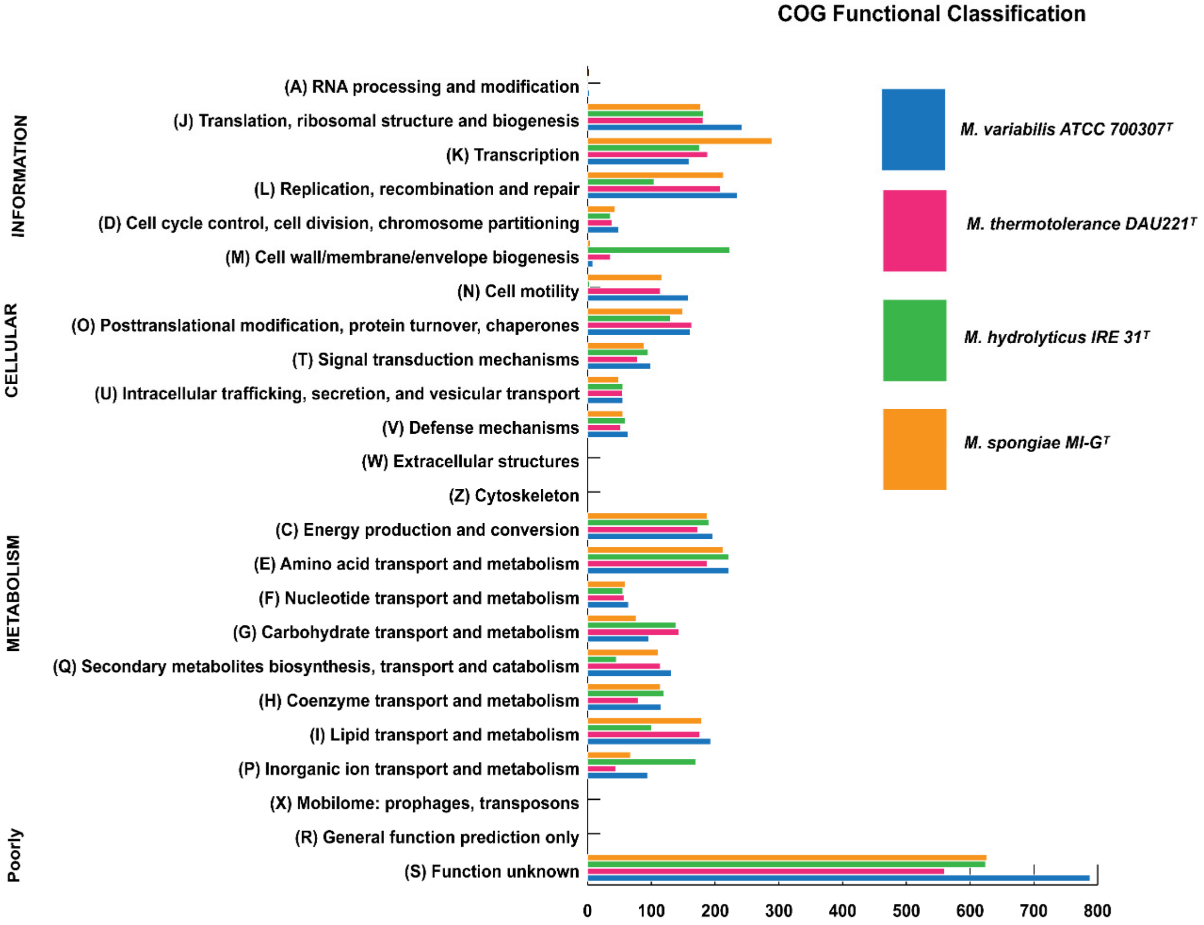

Figure S4. COG functional distribution of gene families in genome of *M. spongiae* MI-G^{T}, and three reference *Microbulbifer* strains *M. hydrolyticus* IRE31^{T}, *M. thermotolerance* DAU221^{T}, and *M. variabilis* ATCC700307^{T}.

http://eggnog-mapper.embl.de/job_status?jobname=MM_awyud2iq

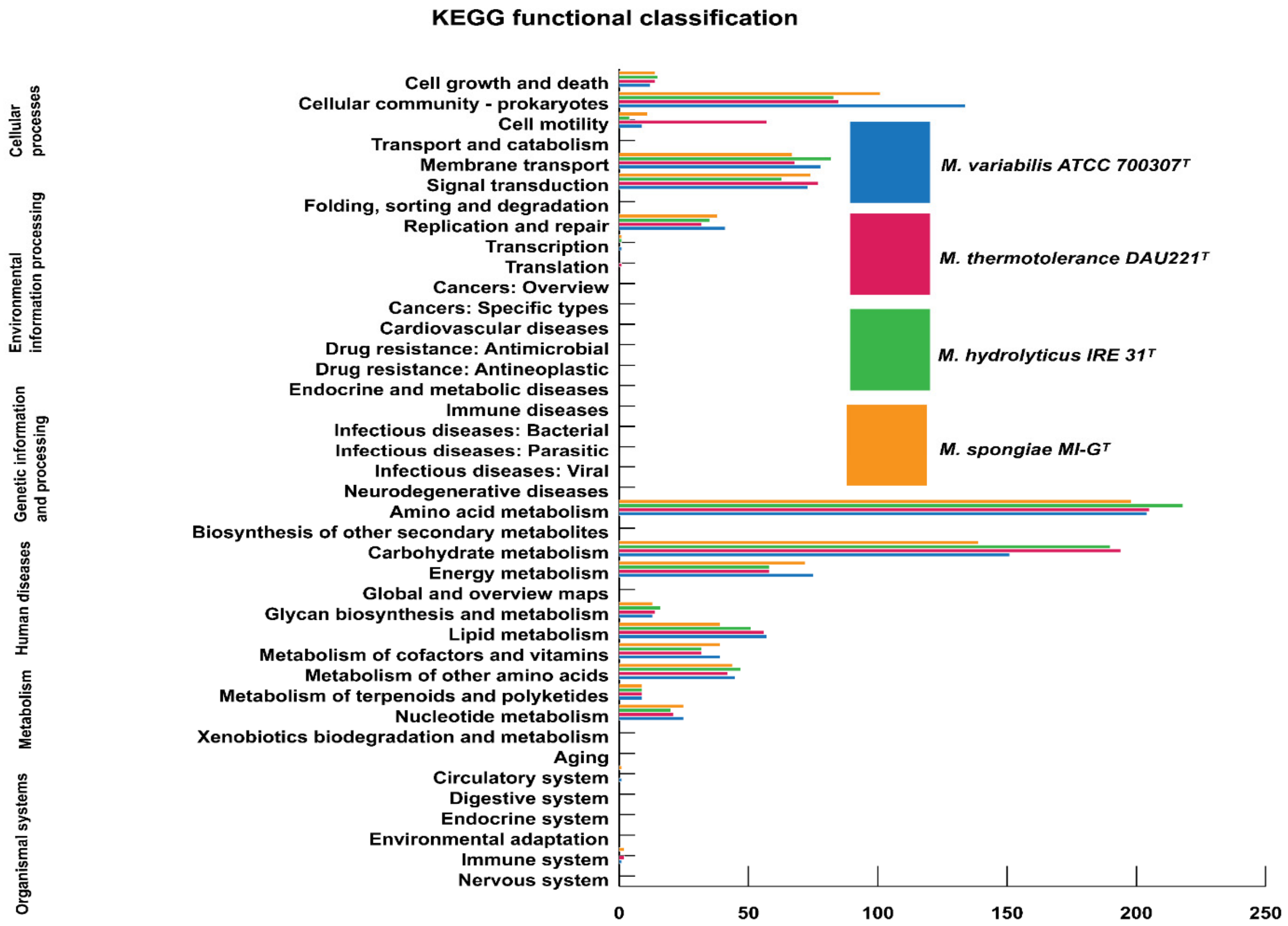

Figure S5. KEGG functional distribution of gene families in genome of *M. spongiae* MI-G^{T}, and three *Microbulbifer* reference strains *M. hydrolyticus* IRE31^{T}, *M. thermotolerance* DAU221^{T}, and *M. variabilis* ATCC700307^{T}.

http://eggnog-mapper.embl.de/job_status?jobname=MM_awyud2iq

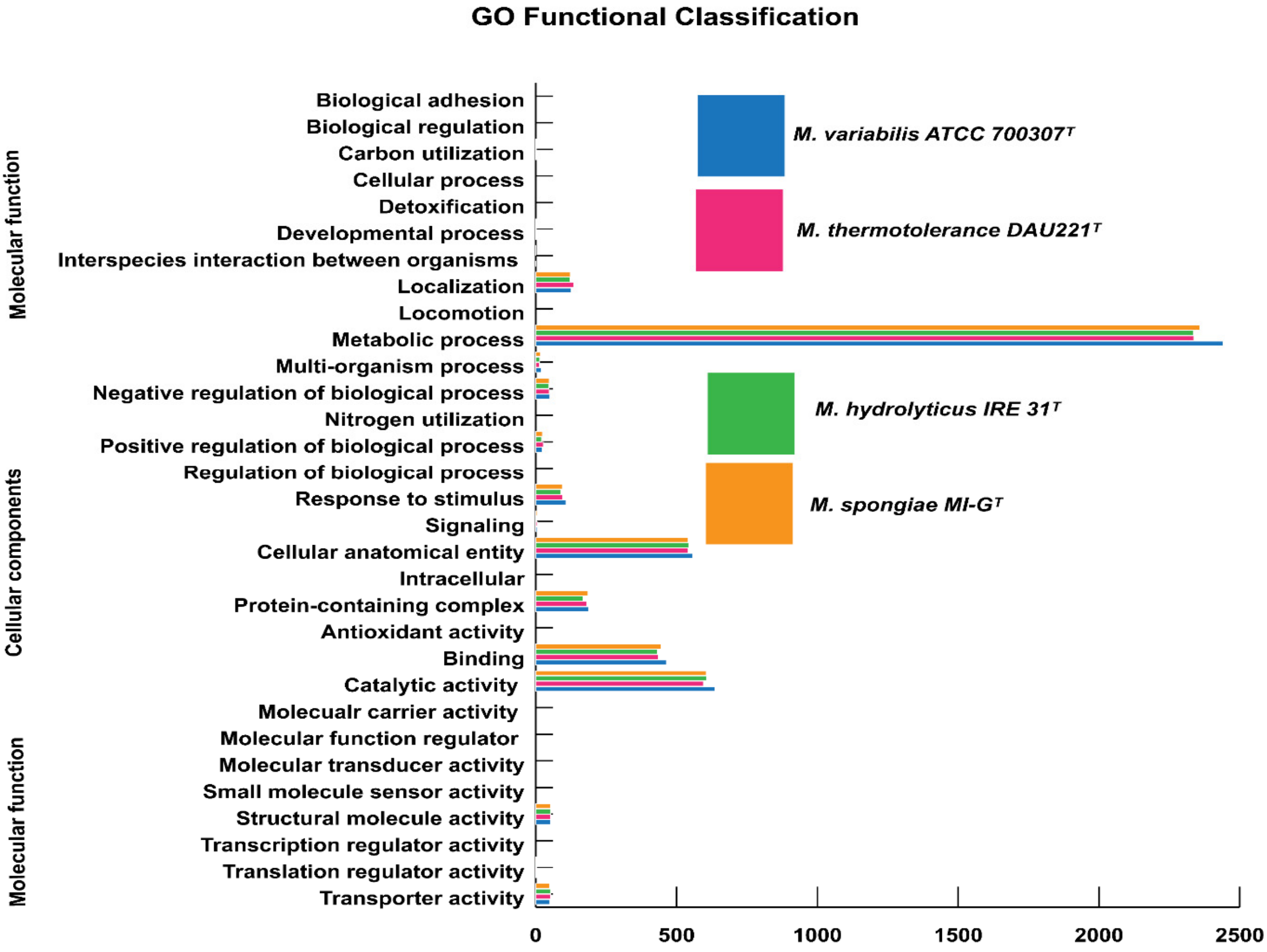

Figure S6. GO functional distribution of gene families in genome of *M. spongiae* MI-G^T, and three *Microbulbifer* reference strains *M. hydrolyticus* IRE31^T, *M. thermotolerance* DAU221^T, and *M. variabilis* ATCC700307^T.

http://eggnog-mapper.embl.de/job_status?jobname=MM_awyud2iq

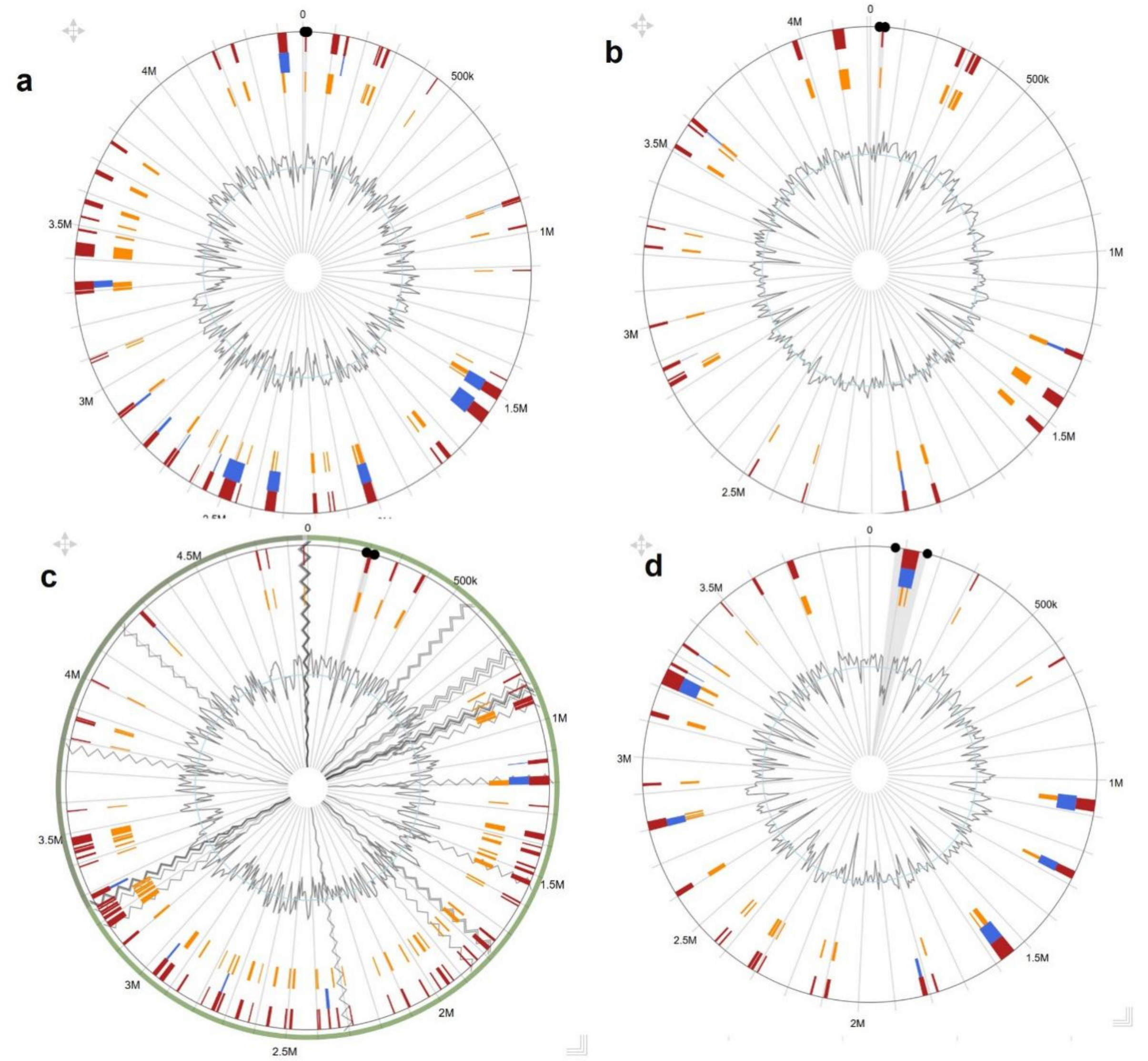

Figure S7. Computational identification and visualization of genomic islands (GIs) in (a) *M. spongiae* MI-G^{T} and three *Microbulbifer* reference strains (b) *M. hydrolyticus* IRE31^{T}, (c) *M. thermotolerance* DAU221^{T}, and (d) *M. variabilis* ATCC700307^{T}.

- Island viewer 4 (https://www.pathogenomics.sfu.ca/islandviewer/)

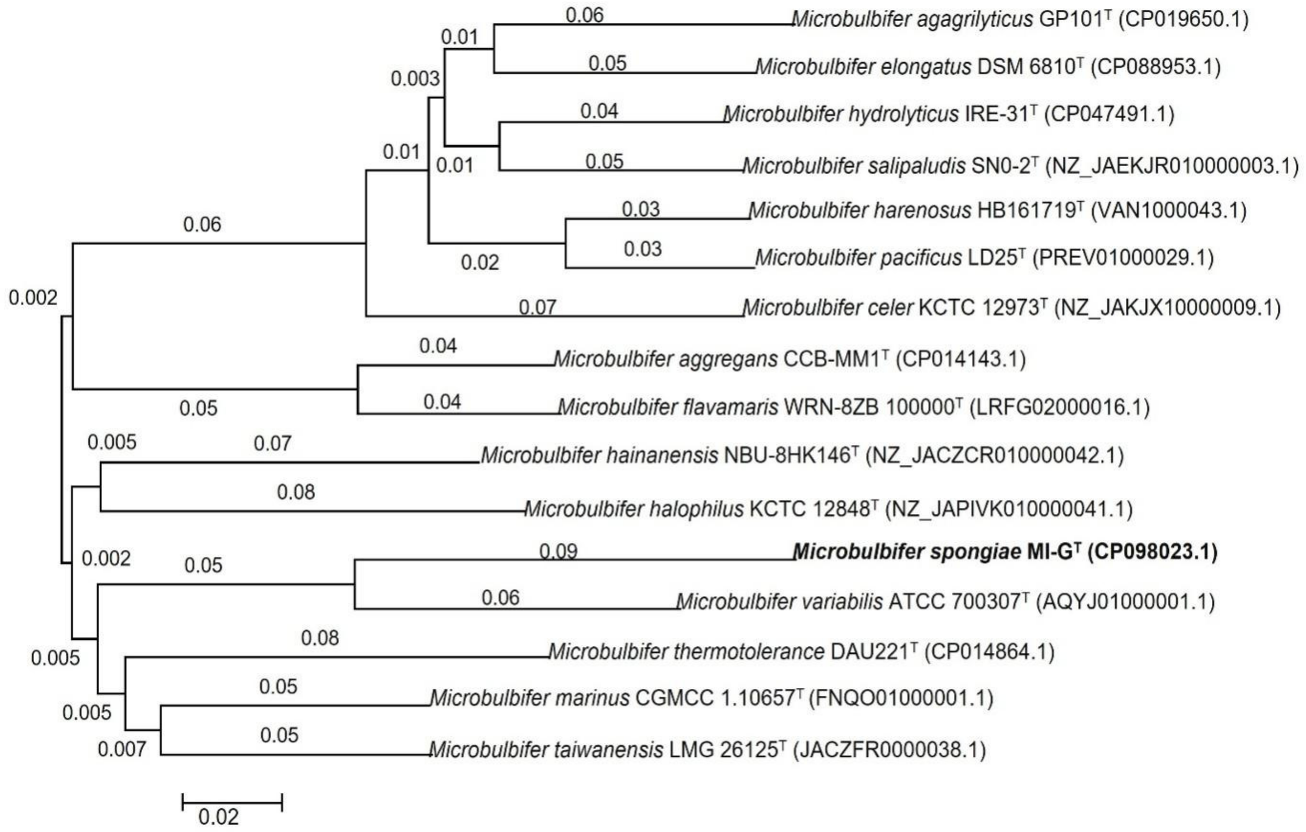

Figure S8. Neighbor-joining (NL) tree based on single-copy orthologous protein sequences of *M. spongiae* MI-G^T and three *Microbulbifer* reference strains. Amino acid sequences were identified using Proteinortho version 6.0 software with cut-off criteria of e-value 1e-5, sequence identity 50%, and sequence coverage 50%. NL tree was calculated with number of bootstrap 1000 replicates. Bar, 0.02 represent substitution per amino acid sequence.

- Software, v6.3.4
- MEGA version 11.0

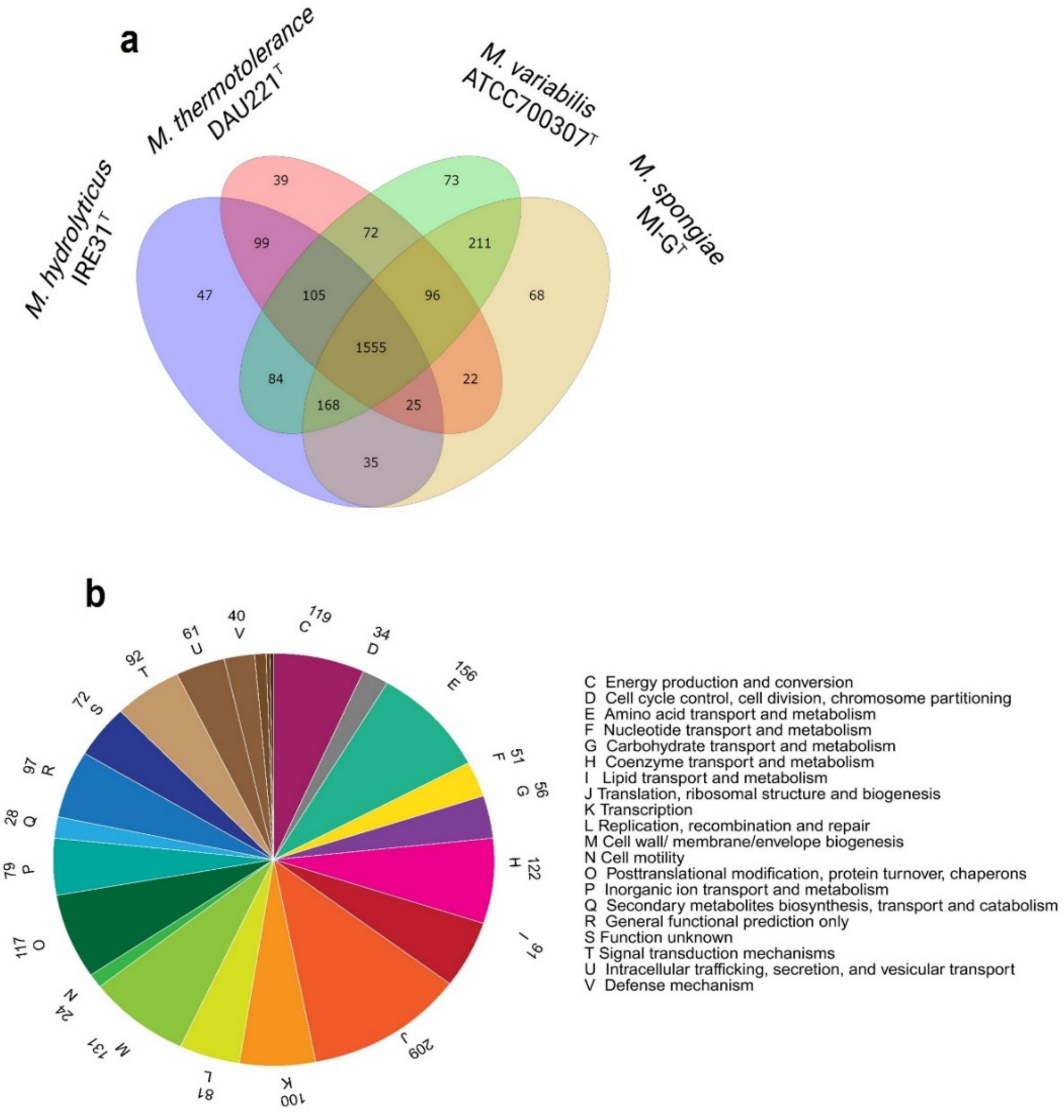

Figure S9. Comparative analysis of *M. spongiae* MI-G^{T} with three reference *Microbulbifer* strains (*M. hydrolyticus* IRE31^{T}, *M. thermotolerance* DAU221^{T} and *M. variabilis* ATCC700307^{T}). (a) Venn diagram showing shared and unique gene family number among *Microbulbifer* strains: *M. spongiae* MI-G^{T}, *M. hydrolyticus* IRE-31^{T}, *M. variabilis* ATCC700307^{T} and *M. thermotolerance* DAU221^{T}. (b) COG functional distribution of gene families in core genome of four *Microbulbifer* strains.

- R program version 4.3.1

Table S1: Stress responsive genes and their respective annotations in the genome of *M. spongiae* MI-G^T and three *Microbulbifer* reference strains

Classification of environment adaptation factors	Adaptation factors	KO_ID	Description	*M. spongiae* MI-G^T	*M. hydrolyticus* IRE31^T	*M. thermotolerance* DAU221^T	*M. variabilis* ATCC700307^T
Oxidative stress response	Superoxide dismutase	K04565<br>K04564	Superoxide dismutase, (1.15.1.1)<br>Superoxide dismutase, Fe-Mn (1.15.1.1)	sodB<br>-	sodB<br>sodC	sodB<br>-	sodB<br>-
	Catalase	K03781<br>K03781<br>K03782<br>-	Catalase (1.11.1.6)<br>Protection from H_2O_2 (1.11.1.6)<br>Catalase-peroxidase (1.11.1.21)<br>Cation efflux family	-<br>katB<br>katG<br>-	katA<br>-<br>katG<br>catA	katA<br>-<br>katG<br>-	-<br>katB<br>katG<br>-
	Peroxireduction	K03564<br>K03386<br>K03387	Peroxiredoxin Q/BCP (1.11.1.15)<br>Alkyl hydroperoxide reductase (1.11.1.15)<br>Alkyl hydroperoxide reductase, large subunit	-<br>ahpC<br>ahpF	bcp<br>ahpC<br>ahpF	-<br>ahpC<br>ahpF	-<br>ahpC<br>ahpF
	Dioxygenase	K00457<br>K00453<br>K00451	4-hydroxyphenylpyruvate dioxygenase<br>Tryptophan 2,3-dioxygenase (1.13.11.11)<br>Homogentisate 1,2-dioxygenase (1.13.11.5)	hppD<br>kynA<br>hmgA	hppD<br>kynA<br>-	hppD<br>kynA<br>-	hppD<br>kynA<br>hmgA
	Cold and heat shock	K00557<br>K03439<br>K00554<br>K00556<br>K02533<br>K03216<br>K03704<br>K03704<br>K04083<br>K04762<br>K03686<br>K04043	Methyltransferase catalysis (2.1.1.35)<br>N (7)-methylguanine catalysis (2.1.1.33)<br>RNA methyltransferase (2.1.1.228)<br>2'-O methylation of guanosine (2.1.1.34)<br>2'O-methylated cytidine (Cm32) (2.1.1.200)<br>Methylates the ribose (2.1.1.207)<br>Cold shock proteins<br>Cold shock proteins<br>Redox regulated molecular chaperone<br>Ribosome-associated heat shock protein<br>ATP binding to DnaK<br>Heat shock 70 kDa protein	trmA<br>trmB<br>trmD<br>-<br>trmJ<br>trmL<br>-<br>cspD<br>hslR<br>dnaJ<br>dnaK<br>dnaN	trmA<br>trmB<br>trmD<br>trmH<br>trmJ<br>trmL<br>cspA<br>cspD<br>hslR<br>dnaJ<br>dnaK<br>dnaN	trmA<br>trmB<br>trmD<br>-<br>trmJ<br>trmL<br>-<br>cspD<br>hslR<br>dnaJ<br>dnaK<br>dnaN	trmA<br>trmB<br>trmD<br>-<br>trmJ<br>trmL<br>cspA<br>cspD<br>hslR<br>dnaJ<br>dnaK<br>dnaN
	Glycine betaine and choline	K00901<br>K00108<br>K00130<br>K02168	Diacylglycerol kinase (ATP) (2.7.1.107)<br>Choline dehydrogenase (1.1.99.1)<br>Biosynthesis of glycine betaine (1.2.1.8)<br>BCCT transporter (TC 2.A.15) family	dgkA<br>-<br>-<br>-	dgkA<br>-<br>-<br>betT	dgkA<br>betA<br>-<br>betT	dgkA<br>betA<br>betB<br>-
Osmotic	Na+ pump	K05565<br>K05566<br>K05568<br>K05569	Multisubunit Na H antiporter, MnhA subunit<br>Na H antiporter, MnhB<br>Multisubunit Na H antiporter, MnhD	mnhA<br>mnhB<br>mnhD<br>-	-<br>-<br>-<br>-	-<br>-<br>-<br>-	mnhA<br>mnhB<br>mnhD<br>mrpE

stress		K05569	subunit	-	mnhE	-	-
response		K05570	Multisubunit Na H antiporter, MnhE	-	mnhF	-	-
		K03313	subunit	nhaA	nhaA	nhaA	nhaA
		K03314	Na+/H+ ion antiporter subunit	-	nhaB	nhaB	nhaB
		K03315	Multiple resistance protein F (MrpF /	nhaC	nhaC	nhaC	nhaC
		K03316	PhaF)	nhaP	nhaP	nhaP	nhaP
			(H) antiporter that extrudes sodium				
			(Na:H) antiporter that extrudes sodium				
			Na:H antiporter				
			Sodium/hydrogen exchanger family				
		K04561	Nitric oxide reductase (1.7.2.5)	norB	-	norB	-
		K03327	Na -driven multidrug efflux pump	norM	norM	norM	norM
		K12266	Transcriptional regulator	-	-	-	norR
Resistance to	-	K03327	Na -driven multidrug efflux pump	dinF	dinF	dinF	dinF
antimicrobia		K03585	Membrane fusion protein (MFP)	acrA	acrA	acrA	acrA
l drugs		K18138	Efflux pump	acrB	acrB	acrB	acrB
		K18138	Resistance-nodulation-cell division	acrF	acrF	-	-
		K03577	(RND)	acrR	acrR	-	acrR
		K18138	MAATS-type transcriptional repressor	mtrD_1	mtrD_1	mtrD_1	mtrD_1
		K03673	Resistance-nodulation-cell division	dsbA	dsbA	dsbA	dsbA
		K03611	(RND)	-	-	dsbB	dsbB
			Thiol disulfide interchange protein				
			Disulfide bond in periplasmic proteins				
		K03805	Disulfide bond in periplasmic proteins	-	-	dsbG	-
		K01467	Beta-lactamase (3.5.2.6)	ampC	ampC	ampC	ampC
		K01447	Beta-lactamase expression (3.5.1.28)	ampD	ampD	ampD	ampD
		K03807	Beta-lactamase induction	ampE	ampE	ampE	ampE
Tolerance to	-	K08218	Acetyl-coenzyme A transporter 1	-	ampG	ampG	ampG
biotoxic		K01262	Creatinase/Prolidase (3.4.11.9)	ampP	ampP	ampP	ampP
metals		K01448	N-acetylmuramoyl-L-alanine (3.5.1.28)	amiC	amiC	amiC	amiC
		K01447	N-acetylmuramoyl-L-alanine	-	amiD_2	amiD_2	-
		K15726	AcrB/AcrD/AcrF family	-	czcA	-	czcA
		K15727	Membrane-fusion protein	-	czcB	-	czcB
		K15725	Outer membrane efflux protein	czcC	-	-	czcC
		K03284	Mediates influx of magnesium ions	-	-	corA	-
		K06189	Mg2 and Co2 transporter CorC	corC	corC	corC	corC
		K04333	Helix_turn_helix, Lux Regulon	corR	-	-	corR

Table S2. Genomic island (GIs) statistics in *M. spongiae* MI-G^T and three *Microbulbifer* reference strains

Strain	ID	Method	Island start	Island end	Length (bp)
	GEI-1	IslandPath-DIMOB	137598	146418	8820
	GEI-2	IslandPath-DIMOB	888541	898661	10120
	GEI-3	IslandPath-DIMOB	1484946	1516845	31899
	GEI-4	IslandPath-DIMOB	1559029	1599200	40171
	GEI-5	IslandPath-DIMOB	2002989	2032028	29039
	GEI-6	IslandPath-DIMOB	2326661	2358542	31881
	GEI-7	IslandPath-DIMOB	2467974	2510309	42335
	GEI-8	IslandPath-DIMOB	2550062	2559264	9202
	GEI-9	IslandPath-DIMOB	2685909	2695024	9115
	GEI-10	IslandPath-DIMOB	2776299	2792405	16106
	GEI-11	IslandPath-DIMOB	2895523	2902492	6969
	GEI-12	IslandPath-DIMOB	3309654	3331813	22159
	GEI-13	IslandPath-DIMOB	4398631	4427283	28652
	GEI-14	SIGI-HMM	7324	12397	5073
	GEI-15	SIGI-HMM	94394	117046	22652
	GEI-14	SIGI-HMM	243949	248009	4060
	GEI-15	SIGI-HMM	250574	258141	7567
	GEI-16	SIGI-HMM	269627	280327	10700
	GEI-17	SIGI-HMM	447490	451757	4267
	GEI-18	SIGI-HMM	892196	896431	4235
	GEI-19	SIGI-HMM	899938	905515	5577
	GEI-20	SIGI-HMM	975040	982071	7031
	GEI-21	SIGI-HMM	1110756	1114889	4133
M. spongiae MI-G^T	GEI-22	SIGI-HMM	1448401	1452776	4375
	GEI-23	SIGI-HMM	1483027	1498224	15197
	GEI-24	SIGI-HMM	1506120	1510837	4717
	GEI-25	SIGI-HMM	1734806	1750454	15648
	GEI-26	SIGI-HMM	1769081	1774782	5701
	GEI-27	SIGI-HMM	2002989	2019988	16999
	GEI-28	SIGI-HMM	2026423	2033930	7507
	GEI-29	SIGI-HMM	2136151	2140970	4819
	GEI-30	SIGI-HMM	2150324	2154504	4180
	GEI-31	SIGI-HMM	2192828	2206099	13271
	GEI-32	SIGI-HMM	2340598	2344637	4039
	GEI-33	SIGI-HMM	2352139	2359279	7140
	GEI-34	SIGI-HMM	2449421	2453703	4282
	GEI-35	SIGI-HMM	2490656	2495595	4939
	GEI-36	SIGI-HMM	2558845	2564799	5954
	GEI-37	SIGI-HMM	2606093	2610252	4159
	GEI-38	SIGI-HMM	2697796	2707736	9940
	GEI-39	SIGI-HMM	2904152	2913317	9165
	GEI-40	SIGI-HMM	3082238	3086445	4207

	GEI-41	SIGI-HMM	3293169	3303204	10035
	GEI-42	SIGI-HMM	3305601	3323759	18158
	GEI-43	SIGI-HMM	3409128	3449913	40785
	GEI-44	SIGI-HMM	3483471	3490899	7428
	GEI-45	SIGI-HMM	3523717	3530012	6295
	GEI-46	SIGI-HMM	3565813	3580883	15070
	GEI-47	SIGI-HMM	3660715	3675646	14931
	GEI-48	SIGI-HMM	3762700	3773340	10640
	GEI-49	SIGI-HMM	4185956	4194446	8490
	GEI-50	SIGI-HMM	4247677	4258577	10900
	GEI-51	SIGI-HMM	4398066	4408723	10657
M. hydrolyticus IRE31^T	GEI-1	IslandPath-DIMOB	1290750	1306039	15289
	GEI-2	IslandPath-DIMOB	1989399	2003801	14402
	GEI-3	IslandPath-DIMOB	3598551	3610944	12393
	GEI-4	SIGI-HMM	34303	39959	5656
	GEI-5	SIGI-HMM	277870	290124	12254
	GEI-6	SIGI-HMM	314153	323350	9197
	GEI-7	SIGI-HMM	327518	342238	14720
	GEI-8	SIGI-HMM	1290009	1306039	16030
	GEI-9	SIGI-HMM	1421897	1455494	33597
	GEI-10	SIGI-HMM	1522683	1542870	20187
	GEI-11	SIGI-HMM	1893553	1907652	14099
	GEI-12	SIGI-HMM	1992989	2003801	10812
	GEI-13	SIGI-HMM	2307044	2312013	4969
	GEI-14	SIGI-HMM	2479399	2485366	5967
	GEI-15	SIGI-HMM	2822004	2834595	12591
	GEI-16	SIGI-HMM	2839360	2844034	4674
	GEI-17	SIGI-HMM	2995346	3003494	8148
	GEI-18	SIGI-HMM	3219622	3227344	7722
	GEI-19	SIGI-HMM	3277302	3282550	5248
	GEI-20	SIGI-HMM	3510271	3523157	12886
	GEI-21	SIGI-HMM	3583459	3589129	5670
	GEI-22	SIGI-HMM	3601890	3610944	9054
	GEI-23	SIGI-HMM	3973066	3989274	16208
	GEI-24	SIGI-HMM	4095926	4130989	35063
M. thermotolerance DAU221^T	GEI-1	IslandPath-DIMOB	1122422	1135863	13441
	GEI-2	IslandPath-DIMOB	1178022	1204652	26630
	GEI-3	IslandPath-DIMOB	2356250	2362716	6466
	GEI-4	IslandPath-DIMOB	2737954	2746920	8966
	GEI-5	IslandPath-DIMOB	2956614	2972585	15971
	GEI-6	IslandPath-DIMOB	3276779	3293131	16352
	GEI-7	IslandPath-DIMOB	4267871	4281812	13941
	GEI-8	SIGI-HMM	197144	210109	12965
	GEI-9	SIGI-HMM	289650	297692	8042

	GEI-10	SIGI-HMM	382902	394669	11767
	GEI-11	SIGI-HMM	931510	942047	10537
	GEI-12	SIGI-HMM	1187307	1207890	20583
	GEI-13	SIGI-HMM	1365932	1386242	20310
	GEI-14	SIGI-HMM	1275212	1280293	5081
	GEI-15	SIGI-HMM	1365932	1386242	20310
	GEI-16	SIGI-HMM	1426918	1439987	13069
	GEI-17	SIGI-HMM	1476462	1482867	6405
	GEI-18	SIGI-HMM	1521844	1538142	16298
	GEI-19	SIGI-HMM	1596895	1603044	6149
	GEI-20	SIGI-HMM	1609204	1614170	4966
	GEI-21	SIGI-HMM	1769967	1785452	15485
	GEI-22	SIGI-HMM	1855458	1861177	5719
	GEI-23	SIGI-HMM	1976425	1981522	5097
	GEI-24	SIGI-HMM	2021220	2027443	6223
	GEI-25	SIGI-HMM	2090201	2098665	8464
	GEI-26	SIGI-HMM	2144846	2149801	4955
	GEI-27	SIGI-HMM	2283359	2287704	4345
	GEI-28	SIGI-HMM	2326793	2332903	6110
	GEI-29	SIGI-HMM	2396302	2400564	4262
	GEI-30	SIGI-HMM	2482497	2491933	9436
	GEI-31	SIGI-HMM	2556077	2566540	10463
	GEI-32	SIGI-HMM	2621615	2625877	4262
	GEI-33	SIGI-HMM	2657323	2669367	12044
	GEI-34	SIGI-HMM	2726240	2733426	7186
	GEI-35	SIGI-HMM	2869483	2876961	7478
	GEI-36	SIGI-HMM	2927890	2945551	17661
	GEI-37	SIGI-HMM	3097777	3107908	10131
	GEI-38	SIGI-HMM	3200387	3211691	11304
	GEI-39	SIGI-HMM	3216665	3223699	7034
	GEI-40	SIGI-HMM	3224685	3232162	7477
	GEI-41	SIGI-HMM	3385927	3390894	4967
	GEI-42	SIGI-HMM	3433088	3443462	10374
	GEI-43	SIGI-HMM	3445672	3452220	6548
	GEI-44	SIGI-HMM	3459324	3486427	27103
	GEI-45	SIGI-HMM	3568831	3573097	4266
	GEI-46	SIGI-HMM	3863351	3871197	7846
	GEI-47	SIGI-HMM	3872260	3878350	6090
	GEI-48	SIGI-HMM	4002276	4007848	5572
	GEI-49	SIGI-HMM	4727083	4731625	4542
	GEI-50	SIGI-HMM	4694713	4701589	6876
	GEI-51	SIGI-HMM	4727083	4731625	4542
	GEI-52	SIGI-HMM	4848754	4852911	4157
	GEI-1	IslandPath-DIMOB	93698	138625	44927

M. variabilis ATCC 700307^T	GEI-2	IslandPath-DIMOB	1055364	1088258	32894
	GEI-3	IslandPath-DIMOB	1259846	1283217	23371
	GEI-4	IslandPath-DIMOB	1536796	1582010	45214
	GEI-5	IslandPath-DIMOB	1806151	1820889	14738
	GEI-6	IslandPath-DIMOB	2796740	2817843	21103
	GEI-7	IslandPath-DIMOB	3209538	3254087	44549
	GEI-8	IslandPath-DIMOB	3267591	3276129	8538
	GEI-9	IslandPath-DIMOB	3330199	3338556	8357
	GEI-10	IslandPath-DIMOB	3596696	3606210	9514
	GEI-11	SIGI-HMM	123989	129063	5074
	GEI-12	SIGI-HMM	311380	315973	4593
	GEI-13	SIGI-HMM	643084	650231	7147
	GEI-14	SIGI-HMM	1055364	1071415	16051
	GEI-15	SIGI-HMM	1265685	1276193	10508
	GEI-16	SIGI-HMM	1545190	1562731	17541
	GEI-17	SIGI-HMM	1573600	1578490	4890
	GEI-18	SIGI-HMM	2086586	2097120	10534
	GEI-19	SIGI-HMM	2130402	2136805	6403
	GEI-20	SIGI-HMM	2291342	2295850	4508
	GEI-21	SIGI-HMM	2306590	2315232	8642
	GEI-22	SIGI-HMM	2316830	2325659	8829
	GEI-23	SIGI-HMM	2420670	2425536	4866
	GEI-24	SIGI-HMM	2435103	2440880	5777
	GEI-25	SIGI-HMM	2597738	2613279	15541
	GEI-26	SIGI-HMM	2801051	2806155	5104
	GEI-27	SIGI-HMM	2807835	2812600	4765
	GEI-28	SIGI-HMM	2817010	2822788	5778
	GEI-29	SIGI-HMM	2921237	2929293	8056
	GEI-30	SIGI-HMM	3117590	3131795	14205
	GEI-31	SIGI-HMM	3209538	3215495	5957
	GEI-32	SIGI-HMM	3244680	3255823	11143
	GEI-33	SIGI-HMM	3331458	3338556	7098
	GEI-34	SIGI-HMM	3487881	3492266	4385
	GEI-35	SIGI-HMM	3703405	3722713	19308

Table S3. Insertion sequence (ISs) element statistics in *M. spongiae* MI-G^T and three *Microbulbifer* reference strains

Strains	Contig	Sequences producing significant alignments	ISfamily	Origin	Score (bits)	E-value
	CP098023.1	SRosp5	IS3	*Roseobacter sp.*	315	4.00E-82
	CP098023.1	ISMahy14	IS256	*Marinobacter*	184	9.00E-43
	CP098023.1	ISPpu12	ISL3	*hydrocarbonoclasticus*	165	8.00E-37
	CP098023.1	IS1396	ISL3	*Pseudomonas putida*	153	8.00E-37
	CP098023.1	ISPst9	ISL3	*Serratia marcescens*	153	3.00E-33
	CP098023.1	ISPa127	IS3	*Pseudomonas stutzeri*	133	3.00E-27
	CP098023.1	ISShes11	Tn3	*Pseudomonas aeruginosa*	125	7.00E-25
	CP098023.1	IS222	IS3	*Shewanella sp.*	123	3.00E-24
	CP098023.1	ISPst2	ISL3	*Pseudomonas aeruginosa*	111	1.00E-20
	CP098023.1	ISSba12	IS256	*Pseudomonas stutzeri*	99.6	4.00E-17
	CP098023.1	ISSod5	IS256	*Shewanella baltica*	99.6	4.00E-17
	CP098023.1	ISPmi1	IS3	*Shewanella oneidensis*	95.6	6.00E-16
	CP098023.1	ISPye6	IS3	*Proteus mirabilis*	93.7	2.00E-15
	CP098023.1	ISAs22	IS3	*Paracoccus yeei*	93.7	2.00E-15
	CP098023.1	ISAs6	IS3	*Aeromonas salmonicida*	91.7	1.00E-14
	CP098023.1	ISAs33	IS3	*Aeromonas salmonicida*	85.7	6.00E-13
	CP098023.1	ISAve4	IS3	*Aeromonas salmonicida*	85.7	6.00E-13
	CP098023.1	ISCARN85	IS5	*Aeromonas veronii*	85.7	6.00E-13
	CP098023.1	ISEcl11	IS30	*Metagenomic data*	83.8	2.00E-12
	CP098023.1	ISNha4	IS3	*Nitrobacter hamburgensis*	83.8	2.00E-12
	CP098023.1	ISSod2	IS3	*Shewanella oneidensis*	81.8	9.00E-12
M. spongiae MI-G^T	CP098023.1	ISAeca6	IS3	*Aeromonas caviae*	79.8	4.00E-11
	CP098023.1	ISAeme6	IS3	*Aeromonas media*	77.8	1.00E-10
	CP098023.1	ISKpn10	IS3	*Klebsiella pneumoniae*	77.8	1.00E-10
	CP098023.1	ISOan5	IS256	*Octadecabacter antarcticus*	75.8	6.00E-10
	CP098023.1	ISPmar2	IS3	*Paracoccus marcusii*	75.8	6.00E-10
	CP098023.1	ISAba18	IS3	*Acinetobacter baumannii*	71.9	9.00E-09
	CP098023.1	ISMaq1	IS3	*Marinobacter aquaeolei*	69.9	4.00E-08
	CP098023.1	ISAba66	IS3	*Acinetobacter baumannii*	67.9	1.00E-07
	CP098023.1	ISPmo5	IS3	*Pseudomonas monteilii*	67.9	1.00E-07
	CP098023.1	ISPa126	IS3	*Pseudomonas aeruginosa*	67.9	1.00E-07
	CP098023.1	TnXo19	Tn3	*Xanthomonas oryzae*	67.9	1.00E-07
	CP098023.1	ISAba29	IS3	*Acinetobacter baumannii*	67.9	1.00E-07
	CP098023.1	ISAba19	IS3	*Acinetobacter baumannii*	67.9	1.00E-07
	CP098023.1	ISAjo3	IS3	*Acinetobacter johnsonii*	65.9	6.00E-07
	CP098023.1	ISAba63	IS3	*Acinetobacter baumannii*	65.9	6.00E-07
	CP098023.1	ISAba57	IS3	*Acinetobacter baumannii*	65.9	6.00E-07
	CP098023.1	ISAlw23	IS3	*Acinetobacter lwoffii*	65.9	6.00E-07

	CP098023.1	ISAlw4	IS3	*Acinetobacter lwoffii*	65.9	6.00E-07
	CP098023.1	ISCfr25	IS3	*Citrobacter freundii*	65.9	6.00E-07
	CP098023.1	ISAba34	IS3	*Acinetobacter baumannii*	65.9	6.00E-07
	CP098023.1	ISTdr1	IS256	*Thiorhodococcus drewsii*	65.9	6.00E-07
	CP098023.1	ISSse3	IS3	*Shewanella sediminis*	65.9	6.00E-07
	CP098023.1	ISShfr8	IS3	*Shewanella frigidimarina*	65.9	6.00E-07
	CP098023.1	ISRme4	IS21	*Cupriavidus metallidurans*	65.9	6.00E-07
	CP098023.1	ISOan8	IS256	*Octadecabacter antarcticus*	65.9	2.00E-06
	CP098023.1	ISAfe2	IS256	*Acidithiobacillus*	63.9	2.00E-06
	CP098023.1	ISAca1	IS3	*ferrooxidans*	63.9	2.00E-06
	CP098023.1	ISAba58	IS256	*Acinetobacter calcoaceticus*	63.9	9.00E-06
	CP098023.1	ISNarch6	IS66	*Acinetobacter baumannii*	61.9	9.00E-06
	CP098023.1	ISOan6	IS256	*Natrialbaceae archaeon*	61.9	9.00E-06
	CP098023.1	ISEca11	IS3	*Octadecabacter antarcticus*	61.9	9.00E-06
	CP098023.1	ISKpn53	IS3	*Erwinia carotovora*	61.9	3.00E-05
	CP098023.1	ISAbe6	IS3	*Klebsiella pneumoniae*	60	3.00E-05
	CP098023.1	ISXac2	IS3	*Acinetobacter bereziniae*	60	3.00E-05
	CP098023.1	ISAba2	IS3	*Xanthomonas axonopodis*	60	3.00E-05
	CP098023.1	ISPsy13	IS3	*Acinetobacter baumannii*	60	3.00E-05
	CP098023.1	IS868	IS3	*Pseudomonas syringae*	60	3.00E-05
	CP098023.1	ISEc93	IS481	*Agrobacterium tumefaciens*	60	1.00E-04
	CP098023.1	ISSma12	IS3	*Escherichia coli*	58	1.00E-04
	CP098023.1	ISAlw5	IS3	*Serratia marcescens*	58	1.00E-04
	CP098023.1	ISEcl10	IS3	*Acinetobacter lwoffii*	58	1.00E-04
	CP098023.1	ISPpu33	IS3	*Enterobacter cloacae*	58	1.00E-04
	CP098023.1	ISPa195	IS3	*Pseudomonas putida*	58	1.00E-04
	CP098023.1	ISPye36	IS3	*Pseudomonas aeruginosa*	58	1.00E-04
	CP098023.1	ISEc31	IS3	*Paracoccus yeei*	58	1.00E-04
	CP098023.1	ISShfr6	IS481	*Escherichia coli*	58	1.00E-04
	CP098023.1	ISShes15	IS3	*Shewanella frigidimarina*	58	1.00E-04
	CP098023.1	ISSba5	IS3	*Shewanella sp.*	58	1.00E-04
	CP098023.1	ISRme15	IS3	*Shewanella baltica*	58	1.00E-04
	CP098023.1	IS51	IS3	*Ralstonia metallidurans*	58	1.00E-04
	CP098023.1	IS1051	IS1595	*Pseudomonas syringae*	58	1.00E-04
M. hydrolyticus IRE 31[T]	CP047491.1	TnAs3	Tn3	*Aeromonas salmonicida*	85.7	6.00E-13
	CP047491.1	ISPa42	IS481	Pseudomonas aeruginosa	81.8	9.00E-12
	CP047491.1	ISRe46	ISNCY	*Rhodococcus equi*	60	3.00E-05
	CP047491.1	ISArsp14	IS3	*Arthrobacter sp.*	60	3.00E-05
	CP047491.1	ISPpu33	IS1634	*Pseudomonas putida*	58	1.00E-04
	CP047491.1	ISVsp2	IS1634	*Verrucomicrobium spinosum*	58	1.00E-04
	CP047491.1	ISSpu18	IS481	*Shewanella putrefaciens*	58	1.00E-04
	CP047491.1	ISXa1	ISAS1	*Xanthobacter autotrophicus*	58	1.00E-04
	CP047491.1	ISAnsp5	IS21	*Anaeromyxobacter sp.*	56	5.00E-04
	CP047491.1	ISAzs17	Tn3	*Azospirillum sp.*	54	0.002

M. thermotolerance DAU221^T	CP014864.1	ISSba12	IS256	*Shewanella baltica*	155	7.00E-34
	CP014864.1	ISEcl11	IS30	*Enterobacter cloacae*	143	3.00E-30
	CP014864.1	ISSod5	IS256	*Shewanella oneidensis*	101	9.00E-18
	CP014864.1	TnXax1	Tn3	*Xanthomonas axonopodis*	87.7	1.00E-13
	CP014864.1	ISSde5	IS256	*Shewanella denitrificans*	79.8	3.00E-11
	CP014864.1	ISPst2	ISL3	*Pseudomonas stutzeri*	79.8	3.00E-11
	CP014864.1	ISSen4	IS3	*Salmonella enterica*	73.8	2.00E-09
	CP014864.1	ISSpr2	IS3	*Serratia proteamaculans*	73.8	2.00E-09
	CP014864.1	ISYen3	IS3	*Yersinia enterocolitica*	73.8	2.00E-09
	CP014864.1	IS1222	IS3	*Enterobacter agglomerans*	73.8	2.00E-09
	CP014864.1	ISPa42	Tn3	*Pseudomonas aeruginosa*	67.9	1.00E-07
	CP014864.1	ISRaq1	IS3	*Rahnella aquatilis*	65.9	5.00E-07
	CP014864.1	ISPsy45	IS5	*Pseudomonas syringae*	63.9	2.00E-06
	CP014864.1	ISKpn78	IS3	*Klebsiella pneumoniae*	60	3.00E-05
	CP014864.1	ISPha2	IS5	*Paracoccus halophilus*	60	3.00E-05
	CP014864.1	ISKpn34	IS3	*Klebsiella pneumoniae*	58	1.00E-04
	CP014864.1	ISEcl5	IS5	*Enterobacter cloacae*	58	1.00E-04
	CP014864.1	TnXo19	Tn3	*Xanthomonas oryzae*	56	5.00E-04
	CP014864.1	ISAzs17	Tn3	*Azospirillum sp.*	56	5.00E-04
M. variabilis ATCC700307^T	NZ_AQYJ01000001.1	ISAba58	IS256	*Acinetobacter baumannii*	34.2	0.61

Table S4. Predictive statistics and description of CRISPR in *M. spongiae* MI-G^T and three *Microbulbifer* reference strains

**Strain**	**CRISPR_ID**	**Start**	**End**	**No. spacers**	**Dr_consensus**
M. spongiae MI-G^T	CRISPR_1	3725115	3725224	2	GCTGCTGCAGAGAAAAAGAAAGC
M. hydrolyticus IRE31^T	CRISPR_1	-	-	-	-
M. thermotolerance DAU221^T	CRISPR_1<br>CRISPR_2<br>CRISPR_3<br>CRISPR_4<br>CRISPR_5	748995<br>3535881<br>3537536<br>3539423<br>3824499	752075<br>3536270<br>3537851<br>3540087<br>3824601	50<br>5<br>4<br>9<br>1	CTGTTCCCCGCATGTGCGGGGATAAACCG<br>GTCTTAATCCCTTTCAAATCAGGGCATCTTTCAAAC<br>GTCTTAATCCCTTTCAAATCAGGGCATCTTTCAAAC<br>GTCTTAATCCCTTTCAAATCAGGGCATCTTTCAAAC<br>GGGAGCACGCCGACCACAACATCTA
M. variabilis ATCC 700307^T	CRISPR_1	-	-	-	-

Table S5. Classification of putative carbohydrate-Active enzymes, corresponding coding genes and (annotation), present in the genome of *M. spongiae* MI-G^T and three *Microbulbifer* reference strains

**CAZyme_class**	**Description of known_activities**	**MI-G^T**	**IRE31^T**	**DAU221^T**	**ATCC 700307^T**
**Auxiliary activities (AA)**					
AA0	N/A	1	-	-	-
AA1	p-diphenol: oxygen oxidoreductase	1	-	4	5
AA1_1	Laccase / p-diphenol: oxygen oxidoreductase / ferroxidase	-	-	-	2
AA1_2	Ecdysone oxidase; oligosaccharide dehydrogenase	-	-	-	2
AA1_3	Laccase-like multicopper oxidase	-	-	-	2
AA2	versatile peroxidase	-	1	2	1
AA3	Cellobiose dehydrogenase	2	1	5	4
AA3_1	aryl alcohol oxidase	-	1	1	4
AA3_2	glucose 1-oxidase	2	1	1	4
AA3_3	alcohol oxidase	-	1	1	3
AA3_4	pyranose oxidase	-	1	1	1
AA4	vanillyl-alcohol oxidase	-	1	-	-
AA6	1,4-benzoquinone reductase	1	1	1	2
AA7	Cello-oligosaccharide dehydrogenase	-	2	1	2
AA8	Iron reductase domain	-	1	1	3
AA10	C1-hydroxylating, C4-dehydrogenating	4	2	2	1
AA12	Quinone-dependent oxidoreductase activity	-	2	1	2
**Carbohydrate-binding modules (CBMs)**					
CBM0	CBM not yet assigned to a family	1	-	-	-
CBM2	Cellulose-binding	3	5	8	1
CBM3	Cellulose-binding in many cases	1	1	1	-
CBM5	Chitin-binding	8	2	3	3
CBM6	Cellulose-binding	1	6	8	-
CBM10	Cellulose-binding	1	-	-	-
CBM12	Chitin-binding	2	1	-	1
CBM13	N/A	4	1	2	1
CBM16	Carbohydrate-binding module 16. Binding to cellulose and glucomannan	-	1	3	-
CBM20	Granular starch-binding	1	-	1	-
CBM22	Xylan binding	1	1	-	-

CBM26	Starch-binding function demonstrated in two cases	-	1	-	-
CBM32	Binding to galactose, lactose, polygalacturonic acid, and LacNAc	-	3	6	-
CBM35	Modules of approx. 130 residues	-	5	6	-
CBM44	The C-terminal CBM44 module bind equally well cellulose and xyloglucan	-	2	1	1
CBM47	Modules of approx 150 residues. Fucose-binding activity demonstrated	-	1	1	-
CBM48	Glycogen-binding function	1	1	-	-
CBM50	Enzymes cleaving	5	1	2	1
CBM51	Modules of approx. 150 residues found attached to various enzymes from families GH2, GH27, GH31, GH95, GH98 and GH101	-	-	1	-
CBM56	beta-1,3-glucan binding	-	1	1	1
CBM60	Modules of approx 120 residues, has xylan-binding function	-	1	-	-
CBM73	Chitin-binding	2	3	6	-
**Carbohydrate esterases (CEs)**					
CE0	Carbohydrate-esterases, not assigned to family	1	-	-	-
CE1	cinnamoyl esterase	-	8	5	7
CE4	chitin deacetylase	-	2	-	2
CE6	acetyl xylan esterase	-	-	2	-
CE8	N-acetyl-glucosamine 6-phosphate	1	2	1	-
CE9	N-acetyl-glucosamine 6-phosphate deacetylase	2	5	6	5
CE10	N/A	-	14	4	8
CE11	UDP-3-0-acyl N-acetylglucosamine	1	1	1	1
CE12	pectin acetyl-esterase	-	1	1	-
CE13	pectin acetylesterase	-	-	-	1
**Glycoside hydrolases (GHs)**					
GH0					
GH1	Not yet assigned to a family	4	-	-	-
GH2	N/A	4	-	-	-
GH3	beta-D-galactofuranosidase	-	2	-	-
GH5	alpha-L-arabinofuranosidase	3	6	7	2
GH5_1	Endo-b-1,4-glucanase; lichenase	3	3	4	-
GH5_2	No known activity in this subfamily	1	2	2	-
GH5_5	Endoglucanase; cellobiohydrolase	1	-	1	-
GH5_8	endo-beta-1,6-galactanase	-	2	1	-
GH5_9	endo-beta-1,3-glucanase / laminarinase	-	2	4	-
GH5_11	endo-beta-1,4-glucanase / cellulase	-	2	1	-

GH5_14	glucan beta-1,3-glucosidase	1	-	-	-
GH5_15	lichenase / endo-beta-1,3-1,4-glucanase	-	1	-	-
GH5_20	glucan endo-1,6-beta-glucosidase	-	-	1	-
GH5_22	beta-glycosidase	-	1	1	-
GH5_25	beta-primeverosidase	-	2	1	-
GH5_26	mannan transglycosylase	-	1	1	-
GH5_36	beta-1,3-mannanase	-	-	1	-
	glucomannan-specific, endo-beta-1,4-glucanase	-	2	1	-
GH5_37	beta-mannosidase				
GH5_38	mannan endo-beta-1,4-mannosidase	-	1	1	-
GH5_39	exo-beta-1,4-glucanase / cellodextrinase	-	2	1	-
GH5_44	cellulose beta-1,4-cellobiosidase	-	2	1	-
GH5_45	beta-N-acetyl hexosaminidase	-	2	1	-
GH5_46	chitosanase	-	1	-	-
GH5_47	steryl beta-glucosidase	-	2	1	-
GH5_50	endoglycoceramidase	-	2	1	-
GH5_53	beta-glucosylceramidase	-	2	1	-
GH5_54	beta-galactosylceramidase	-	1	1	-
GH5_55	beta-rutinosidase /alpha-L-rhamnose-(1,6)-beta-	-	2	1	-
	D-glucosidase	-	-	1	-
GH6	Mannosylglycerate hydrolase				
GH9	N/A	1	-	1	-
GH10	endo-beta-1,4-glucanase	1	2	4	-
GH11	exo-1,4-beta-xylosidase	-	3	1	-
GH13	Cyclomaltodextrin lucanotransferase	-	1	-	-
GH13_1	branching enzyme	10	8	9	6
GH13_2	Cyclica-1,6-maltosyl-maltose hydrolase	-	6	7	4
GH13_3	Glucosyl -glycerate phosphorylase	1	7	8	4
GH13_4	Glucosyl glycerol phosphorylase	-	4	4	2
GH13_5	4-alpha-glucanotransferase	-	3	3	1
GH13_6	alpha-1,4-glucan: phosphate alpha-	-	4	4	3
	maltosyltransferase	-	4	5	3
GH13_7	maltopentaose-forming alpha-amylase				
GH13_9	alpha-amylase	-	1	3	1
GH13_10	alpha-amylase	-	3	3	2
GH13_11	Pullulanase; isoamylase	-	8	4	4
GH13_12	maltotriose-forming alpha-amylase	1	4	2	3
GH13_13	Maltopentaose-producing a-amylase	-	9	7	6
GH13_14	Neopullulanase	1	8	8	6
GH13_15	malto-oligosyltrehalose trehalohydrolase	-	9	8	6
GH13_16	alpha-glucosidase	-	-	1	1
GH13_17	amylo-alpha-1,6-glucosidase	-	7	6	2
GH13_18	pullulanase	-	7	6	1
GH13-19	Maltotriose-producing a-amylase	-	3	2	1

GH13_20	a-glucosyltransferase	1	5	5	3
GH13_21	isoamylase	1	8	5	3
GH13_23	Malto-oligosyltrehalose synthase	-	6	6	2
GH13_24	Maltohexaose-forming alpha-amylase	2	7	7	3
GH13_26	a-glucosidase	-	-	1	2
GH13_27	Amylosucrase	1	1	2	-
GH13_28	sucrose 6(F)-phosphate phosphorylase	-	1	1	2
GH13_29	Amylosucrase	-	6	5	5
GH13_30	sucrose phosphorylase	-	6	6	1
GH13_31	sucrose alpha-glucosidase	-	6	7	3
GH13_32	Isomaltose synthase	-	6	6	3
GH13_34	amino acid transporter	-	7	6	6
GH13_35	trehalose-6-phosphate hydrolase	-	1	1	1
GH13_36	N/A	-	4	4	2
GH13_37	N/A	-	7	6	4
GH13_38	Chitinase; lysozyme;	-	8	6	5
GH13_39	N/A	1	1	1	1
GH13_40	N/A	-	6	5	3
GH13_41	N/A	-	6	6	2
GH13_42	N/A	-	9	7	5
GH13_162	N/A	-	4	5	3
GH16	beta-agarase	-	-	1	-
GH18	Chitinase; lysozyme	-	17	5	-
GH19	N/A	3	4	4	3
GH20	Lysozyme type G; peptidoglycan lyase	1	1	-	1
GH23	Lysozyme	1	1	1	1
GH24	Exo-polygalacturonase	6	8	5	6
GH28	Glucosidase; xylan 1,4-xylosidase	1	-	-	-
GH31	Cellulase; endo-1,4-xylanase	4	1	1	-
GH32	levan fructosyltransferase	1	1	2	-
GH35	beta-1,3-galactosidase	-	-	2	-
GH43	alpha-1,2-L-arabinofuranosidase	-	-	2	-
GH43_1	exo-alpha-1,5-L-arabinofuranosidase	-	3	1	-
GH43_2	exo-alpha-1,5-L-arabinanase	-	1	-	-
GH43_3	endo-alpha-1,5-L-arabinanase	-	2	-	-
GH43_4	exo-beta-1,3-galactanase	-	3	3	-
GH43_5	beta-D-galactofuranosidase	-	2	-	-
GH43_8	beta-1,3-xylosidase	-	2	5	-
GH43_9	beta-xylosidase	-	2	1	-
GH43_10	alpha-L-arabinofuranosidase	-	3	1	-
GH43_11	xylanase	-	3	1	-
GH43_12	N/A	-	2	1	-
GH43_13	N/A	-	2	-	-
GH43_14	N/A	-	2	1	-

GH43_15	N/A	-	2	-	-
GH43_16	N/A	-	2	1	-
GH43_17	N/A	-	2	-	-
GH43_19	N/A	-	1	-	-
GH43_24	N/A	-	3	1	-
GH43_26	N/A	-	1	-	-
GH43_28	N/A	-	3	1	-
GH43_29	N/A	-	1	1	-
GH43_30	N/A	-	3	1	-
GH43_31	N/A	-	3	1	-
GH43_32	N/A	-	3	1	-
GH43_33	N/A	-	3	1	-
GH43_34	N/A	-	2	-	-
GH43_35	N/A	-	3	1	-
GH43_36	N/A	-	1	-	-
GH43_37	N/A	-	2	-	-
GH50	beta-agarase	-	1	-	-
GH51	beta-xylosidase	-	-	2	-
GH63	N/A	-	1	-	-
GH65	α-glucan phosphorylase	1	-	-	-
GH67	xylan alpha-1,2-glucuronidase	-	-	1	1
GH76	Endoglucanase; endo -1,3(4)-glucanase	-	1	1	-
GH74	endoglucanase	1	-	-	-
GH81	endo-beta-1,3-glucanase	-	-	2	-
GH84	3-O-(GlcNAc)-L-Ser/Thr beta-N-	-	-	1	-
	acetylglucosaminidase	-	1	-	-
GH86	beta-porphyranase				
GH87	alpha-1,3-glucanase	-	-	3	-
GH94	beta--1,2-oligoglucan phosphorylase	-	2	-	-
GH95	alpha-L-fucosidase	-	1	1	-
GH103	Peptidoglycan lytic transglycosylase	-	-	1	-
GH104	The enzymes in this family display an unusual	4	1	2	2
	mechanism involving NAD+	1	-	-	-
GH105	d-4,5-unsaturated alpha-galacturonidase				
GH108	N-acetylmuramidase	-	2	1	-
GH109	Endo-1,4-polygalactosaminidase	-	1	-	-
GH114	endo-alpha-1,4-polygalactosaminidase	1	-	-	-
		1	-	-	1
**Glycosyl transferase (GTs)**					
GT0					
GT1	GT not yet assigned to a family	3	-	-	-
GT2	UDP-glucuronosyltransferase	1	-	-	-
GT4	Cellulose synthase; chitin synthase	20	12	6	9

GT5	Sucrose-phosphate synthase	15	10	8	9
GT8	N/A	-	5	4	3
GT19	N/A	-	-	-	1
GT25	Lipid-A-disaccharide synthase	1	1	1	1
GT26	N/A	-	-	1	1
GT27	N/A	-	1	-	-
GT28	N/A	-	1	2	1
GT30	1,2-diacylglycerol 3-galactosyltransferase	3	1	1	1
GT41	KDO transferase	1	1	1	1
GT51	N/A	-	4	5	4
GT81	Murein polymerase	4	3	1	2
GT83	N/A	-	1	1	-
GT84	Dodecaprenyl phospho-b-galacturonic acid	1	-	-	-
GT87	N/A	-	-	1	-
GT107	Polyprenol-P-Man: -1,2-Mannosyltransferaase	3	-	-	-
	CMP-KDO: -2,4-KDO transferase	1	1	-	1
**Polysaccharide lyase (PL)**					
PL1	pectin lyase	-	4	3	-
PL1_2	pectate lyase	-	-	1	-
PL1_3	Exo-pectate lyase	-	2	2	-
PL1_4	N/A	-	2	1	-
PL1_5	N/A	-	3	2	-
PL1_6	N/A	-	2	1	-
PL1_7	N/A	-	2	1	-
PL1_8	N/A	-	1	1	-
PL1_9	N/A	-	2	1	-
PL1_10	N/A	-	2	1	-
PL1_11	N/A	-	2	2	-
PL1_12	N/A	-	2	-	-
PL1_13	N/A	-	1	-	-
PL6	poly(alpha-L-guluronate) lyase / G-specific alginate lyase	-	-	2	-
PL6_1	oligoalginate lyase / exo-alginate lyase	-	-	2	-
PL6_2	MG-specific alginate lyase	-	-	2	-
PL6_3	alginate lyase	-	-	2	-
PL7	alpha-L-guluronate lyase / G-specific alginate lyase	-	-	2	-
PL7_1	oligoalginate lyase / exo-alginate lyase	-	-	2	-
PL7_3	poly-(MG)-lyase / MG-specific alginate lyase	-	-	1	-
PL7_4	poly(beta-mannuronate) lyase / M-specific alginate lyase	-	-	1	-
PL7_5	endo-beta-1,4-glucuronan lyase	-	-	2	-
PL10	pectate lyase	-	2	-	-

PL10_1	N/A	-	2	-	-
PL10_2	N/A	-	3	-	-
PL10_3	N/A	-	2	-	-
PL17	oligoalginate lyase	-	2	1	-
PL17_2	alginate lyase	-	1	1	-
PL22	oligogalacturonate lyase / oligogalacturonide lyase	-	2	1	2
PL29	chondroitin-sulfate ABC endolyase	-	1	-	-

- Program: hmmscan version 3.4
- Genome annotation eggnog (http://eggnog-mapper.embl.de/job_status?jobname=MM_awyud2iq)
- dbCANv2 (diamond mode)

Table S6: Comparison of COG functional classes of *M. spongiae* MI-G^T with three *Microbulbifer* reference strains

First class	Second class	Class description	Core genes	Dispensable genes	MI-G^T specific-genes	IRE31^T specific-genes	DAU221^T specific-genes	ATCC 700307^T specific-genes
Information storage and processing	A	RNA processing and modification	1	1	-	-	-	-
	J	Translation, ribosomal structure and biogenesis	209	33	10	22	4	19
	K	Transcription	100	65	4	42	43	67
	L	Replication, recombination and repair	81	20	11	11	11	10
Metabolism	F	Nucleotide transport and metabolism	51	9	2	10	5	7
	C	Energy production and conversion	119	68	24	40	18	24
	G	Carbohydrate transport and metabolism	56	75	17	74	51	26
	H	Coenzyme transport and metabolism	122	39	17	28	12	35
	I	Lipid transport and metabolism	91	54	64	47	29	40
	E	Amino acid transport and metabolism	156	62	32	53	14	26
	P	Inorganic ion transport and metabolism	79	91	28	62	48	27
	Q	Secondary metabolites biosynthesis, transport and catabolism	28	20	25	18	15	23
	X	Mobilome: prophages, transposons	2	11	27	3	2	20
Cellular	O	Posttranslational modification, protein-turnover, chaperones	117	66	14	37	15	26
	D	Cell cycle control, cell division, chromosome partitioning	34	8	33	5	4	7
	N	Cell motility	24	13	5	9	36	3
	M	Cell wall/membrane/envelope biogenesis	131	80	15	71	27	32
	Z	Cytoskeleton	3	-	-	-	-	1
	S	Function unknown	72	57	13	30	21	31
	T	Signal transduction mechanisms	92	49	15	39	20	19
	U	Intracellular trafficking, secretion, and vesicular transport	61	18	4	7	9	4
	V	Defense mechanisms	40	47	15	28	24	23
	W	Extracellular structures	19	14	4	10	4	2
Poorly	R	General function prediction only	97	84	41	67	50	76

- MUMmer version 4.0
- CD-HIT (software v4.8.1)

Table S7. Comparison of metabolic features of *M. spongiae* MI-G^T with three *Microbulbifer* reference strains

**Metabolic features**	**COG-Id**	**Description**	**Core genome**	**Dispensable genome**	**MI-G^T specific genome**	**IRE31^T specific genome**	**DAU221^T specific genome**	**ATCC 700307^T specific genome**
Ankyrin repeat proteins (ANKs)	COG0666	Ankyrin repeat	-	1	2	-	3	-
Tetra-tricopepetide repeat proteins (TPRs)	COG4783	Outer membrane protein *BepA/YfgC*, contains M48	2	3	-	3	-	-
	COG0457	Tetratricopeptide (TPR) repeat	3	-	-	3	-	-
	COG3118	Chaperedoxin *CnoX,* contains thioredoxin-like and TPR-like domains	3	-	-	3	-	-
	COG4941	Predicted RNA polymerase sigma factor, contains C-terminal TPR domain	-	-	-	-	1	-
	COG4976	Predicted methyltransferase, contains TPR repeat	-	-	-	-	-	6
	COG4700	Uncharacterized conserved protein ECs_4300, contains TPR-like domain	-	-	-	-	-	1
	COG5010	Flp pilus assembly protein TadD, contains TPR repeats	4	-	-	-	-	-
	COG2956	Lipopolysaccharide biosynthesis regulator YciM/LapB, contains six TPR domains and a C-terminal metal-binding domain	1	-	-	-	-	-
	COG3071	Uncharacterized protein HemY, contains HemY_N domain and TPR repeats (unrelated to protoporphyrinogen oxidase HemY)	1	-	-	-	-	-
	COG4206	Outer membrane cobalamin receptor protein BtuB	-	6	3	4	-	4
	COG1120	ABC-type cobalamin/Fe3+-siderophores transport system, ATPase component	-	1	-	-	-	1
	COG0368	Cobalamin synthase CobS (adenosylcobinamide-GDP ribazoletransferase)	-	-	-	-	-	1

Vitamin B12 (Cobalamin)	COG0646	Methionine synthase I (cobalamin-dependent), methyltransferase domain	1	-	-	-	-	-
	COG0620	Methionine synthase II (cobalamin-independent)	1	-	-	-	-	-
	COG1429	Cobalamin biosynthesis protein CobN, Mg-chelatase	-	2	-	-	-	-
Vitamin B6 (Pyridoxine)	COG0259	Pyridoxine/pyridoxamine 5'-phosphate oxidase	1	-	-	-	-	1
	COG0854	Pyridoxine 5'-phosphate synthase PdxJ	1	-	-	-	-	-
Vitamin B1 (Thiamine)	COG0611	Thiamine monophosphate kinase	1	-	-	-	-	-
	COG2104	Sulfur carrier protein ThiS (thiamine biosynthesis)	1	-	-	-	-	-
	COG0476	Molybdopterin or thiamine biosynthesis adenylyltransferase	1	-	1	-	-	-
	COG0028	Acetolactate synthase large subunit or other thiamine pyrophosphate-requiring enzyme	2	2	1	1	-	-
	COG0352	Thiamine monophosphate synthase	1	-	-	-	-	-
	COG0510	Thiamine kinase or a related kinase	-	-	-	1	-	-
	COG0819	Aminopyrimidine aminohydrolase TenA (thiamine salvage pathway)	-	-	-	1	-	-
Vitamin B7 (Biotin)	COG0132	Dethiobiotin synthetase	1	-	-	-	-	-
	COG0502	Biotin synthase or related enzyme	1	-	-	-	-	-
	COG1654	Biotin operon repressor	2	-	-	-	-	-
	COG0511	Biotin carboxyl carrier protein	1	-	-	-	-	-
	COG0439	Biotin carboxylase	1	-	-	-	-	-
	COG0307	Riboflavin synthase alpha chain	1	-	-	-	-	
	COG0054	6,7-dimethyl-8-ribityllumazine synthase (Riboflavin synthase beta chain)	1	-	-	-	-	-
	COG1011	FMN and 5-amino-6-(5-phospho-D-ribitylamino)	1	-	4	-	-	-

Vitamin B2 (Riboflavin)		uracil phosphatase YigB, HAD superfamily (riboflavin biosynthesis)						
	COG0117	Riboflavin biosynthesis protein RibD, pyrimidine deaminase domain	2	-	-	-	-	-
	COG3236	N-glycosidase YbiA/RibX (riboflavin biosynthesis, damage control), NADAR superfamily	-	1	-	-	-	1
Type VI secretion system	COG3517	Predicted component TssB of the type VI protein secretion system, VipA/VipB/TssB family	5	-	-	-	-	-
	COG3456	Predicted component of the type VI protein secretion system, contains an FHA domain	3	-	-	-	-	-
	COG3522	Predicted component of the type VI protein secretion system	1	-	-	-	-	-
	COG3518	Predicted component of the type VI protein secretion system	1	-	-	-	-	-
	COG3519	Type VI protein secretion system component VasA	1	-	-	-	-	-
	COG3157	Type VI protein secretion system component Hcp (secreted cytotoxin)	1	-	-	-	-	-
	COG3515	Type VI protein secretion system component TssA1/VasJ/EvfE, contains ImpA_N domain	1	-	-	-	-	-
	COG3455	Type VI protein secretion system component TssL/VasF/DotU	1	-	-	-	-	-
	COG3521	Predicted component of the type VI protein secretion system	2	-	-	-	-	-
	COG3523	Type VI protein secretion system component VasK	1	-	-	-	-	-
	COG3516		1	-	-	-	-	-

	COG3520	Predicted component TssA of the type VI protein secretion system	1	-	-	-	-	-
	COG3501	Predicted component of the type VI protein secretion system	2	-	-	-	-	-
	COG4104	Uncharacterized conserved protein VgrG, implicated in type VI secretion and phage assembly	-	1	-	-	-	-
		Zn-binding Pro-Ala-Ala-Arg (PAAR) domain, involved in Type VI secretion						
Type III secretion system	COG2257	Type III secretion system substrate exporter, FlhB-like	-	-	-	-	1	-
	COG1766	Flagellar biosynthesis/type III secretory pathway M-ring protein FliF/YscJ	-	-	-	-	1	-
				-				
	COG1317	Flagellar biosynthesis/type III secretory pathway protein FliH	-	-	-	-	1	-
	COG1157	Flagellar biosynthesis/type III secretory pathway ATPase FliI	-	-	-	-	1	-
	COG1886	Flagellar motor switch/type III secretory pathway protein FliN	-	-	-	-	2	-
	COG3418	Flagellar biosynthesis/type III secretory pathway chaperone FlgN	-	-	-	-	1	-
Nitrogen regulatory proteins	COG0347	Nitrogen regulatory protein PII	1	-	-	-	-	-
	COG2844	UTP: GlnB (protein PII) uridylyltransferase	1	-	-	-	-	-
	COG3323	PII-like insert in the uncharacterized protein YqfO, YbgI/NIF3 family	1	-	-	-	-	-
	COG1993	PII-like signaling protein	-	-	-	-	1	-
